# The efficacy of chemotherapy is limited by intratumoral senescent cells expressing PD-L2

**DOI:** 10.1038/s43018-023-00712-x

**Published:** 2024-01-24

**Authors:** Selim Chaib, José Alberto López-Domínguez, Marta Lalinde-Gutiérrez, Neus Prats, Ines Marin, Olga Boix, Andrea García-Garijo, Kathleen Meyer, María Isabel Muñoz, Mònica Aguilera, Lidia Mateo, Camille Stephan-Otto Attolini, Susana Llanos, Sandra Pérez-Ramos, Marta Escorihuela, Fatima Al-Shahrour, Timothy P. Cash, Tamara Tchkonia, James L. Kirkland, María Abad, Alena Gros, Joaquín Arribas, Manuel Serrano

**Affiliations:** 1 Institute for Research in Biomedicine, Barcelona Institute of Science and Technology, Barcelona, Spain; 2 Division of General Internal Medicine, Mayo Clinic, Rochester, MN USA; 3 Vall d’Hebron Institute of Oncology, Vall d’Hebron Barcelona Hospital Campus, Barcelona, Spain; 4 Genentech, South San Francisco, CA USA; 5 Cambridge Institute of Science, Altos Labs, Cambridge, UK; 6 DNA Replication Group, Spanish National Cancer Research Center, Madrid, Spain; 7 Bioinformatics Unit, Spanish National Cancer Research Center, Madrid, Spain; 8 Rejuveron Senescence Therapeutics, Zürich, Switzerland; 9 Department of Physiology and Biomedical Engineering, Mayo Clinic, Rochester, MN USA; 10 Cancer Research Program, Hospital del Mar Medical Research Institute, Centro de Investigación Biomédica en Red Cáncer, Barcelona, Spain; 11 Department of Medicine and Life Sciences, Universitat Pompeu Fabra, Barcelona, Spain; 12 Institució Catalana de Recerca i Estudis Avançats, Barcelona, Spain

**Keywords:** Cancer, Senescence

## Abstract

Chemotherapy often generates intratumoral senescent cancer cells that strongly modify the tumor microenvironment, favoring immunosuppression and tumor growth. We discovered, through an unbiased proteomics screen, that the immune checkpoint inhibitor programmed cell death 1 ligand 2 (PD-L2) is highly upregulated upon induction of senescence in different types of cancer cells. PD-L2 is not required for cells to undergo senescence, but it is critical for senescent cells to evade the immune system and persist intratumorally. Indeed, after chemotherapy, PD-L2-deficient senescent cancer cells are rapidly eliminated and tumors do not produce the senescence-associated chemokines CXCL1 and CXCL2. Accordingly, PD-L2-deficient pancreatic tumors fail to recruit myeloid-derived suppressor cells and undergo regression driven by CD8 T cells after chemotherapy. Finally, antibody-mediated blockade of PD-L2 strongly synergizes with chemotherapy causing remission of mammary tumors in mice. The combination of chemotherapy with anti-PD-L2 provides a therapeutic strategy that exploits vulnerabilities arising from therapy-induced senescence.

## Abstract (web summary)

Chaib et al. find that therapy-induced senescent cells have high programmed cell death 1 ligand 2 (PD-L2), contributing to recurrence. They show that PD-L2 blocking combined with chemotherapy is therapeutically beneficial because it reduces senescent cells and immunosuppressive cell recruitment.

## Main

The inhibitory receptor programmed cell death protein 1 (PD-1, encoded by *PDCD1*) and its ligand programmed cell death 1 ligand 1 (PD-L1/B7-H1, encoded by *CD274*) constitute an important immune checkpoint that controls the establishment of immune tolerance and negatively regulates the activity and proliferation of immune cells^1^. They are also key contributors to immune evasion by cancer cells, which frequently overexpress PD-L1 (ref. ^2^). Immunotherapies targeting PD-1 or PD-L1 have been successfully used in the clinic against a broad spectrum of tumors^3–5^, including melanoma and non-small cell lung cancer^6^. An alternative PD-1 ligand, programmed cell death 1 ligand 2 (PD-L2/B7-DC, encoded by *PDCD1LG2*) has received comparatively less attention due to the lower frequency of PD-L2^+^ cancers, compared to PD-L1 (ref. ^7^).

PD-L1 and PD-L2 compete for binding to PD-1, although, interestingly, PD-L2 exhibits a twofold to sixfold higher binding affinity to PD-1 (ref. ^8^). PD-L2 is predominantly expressed by dendritic cells, macrophages and other antigen-presenting cells. Interestingly, PD-L2 is critical for the function of tolerogenic dendritic cells^9^. Additionally, in the context of cancer, cancer-associated fibroblasts can express PD-L2 and contribute to an immunosuppressive tumor microenvironment^10,11^. PD-L2 expression is detectable in a large variety of human cancers, being most prominent in triple-negative breast cancer, and head and neck squamous cell carcinomas^7,12^. There is limited understanding about the biological role of PD-L2 for immune evasion in cancers, although ectopic expression of PD-L2 produces immune evasion through the inhibition of PD-1 (ref. ^13^). In the case of monocytes and macrophages, interferon-γ (IFN-γ) upregulates both PD-L1 and PD-L2, while interleukin-4 (IL-4) selectively upregulates PD-L2 (refs. ^14,15^). However, at present, there is limited information regarding how the levels of PD-L2 are regulated in cancer. Two recent reports found upregulation of PD-L2 in some cancer cell lines in vitro in response to cisplatin^16^ or radiation^17^. Importantly, PD-L2 has another receptor, repulsive guidance molecule B (RGMb), which is only bound by PD-L2 and not by PD-L1 (ref. ^18^). This receptor is expressed by CD8^+^ tumor-infiltrating T cells and inhibits CD8^+^ T cells on binding to PD-L2 (ref. ^19^).

Conventional chemotherapy or radiotherapy is still the most common treatment for solid cancers. DNA damage and other insults associated with these therapies can trigger therapy-induced senescence (TIS) in cancer cells and in other intratumoral cells, such as endothelial cells and fibroblasts^20^. The senescence program, entailing a stable cell cycle arrest, constitutes a cell-intrinsic barrier against oncogene-driven proliferation and transformation. Cellular senescence also involves a prominent secretory activity known as the senescence-associated secretory phenotype (SASP)^21^, transcriptionally regulated by nuclear factor kappa-light-chain-enhancer of activated B cells (NF-κB) and other factors. The SASP is complex and heterogeneous, including pro-inflammatory cytokines, chemokines, matrix remodeling enzymes and growth factors, which result in cell-extrinsic effects known to facilitate tumor growth^22–25^. More specifically, the SASP includes immunosuppressive factors, such as tumor growth factor-β, and chemokines that recruit myeloid-derived suppressive cells (MDSCs)^26–29^. Therefore, while senescence is a cell-intrinsic barrier for cancer cells, it is also pro-tumorigenic through its extrinsic actions on the tumor microenvironment.

Multiple studies have demonstrated that therapies that selectively eliminate senescent cells, known as senolytic therapies, strongly synergize with cancer chemotherapy^20,30,31^. This is the case with senolytic compounds, such as navitoclax, dasatinib and cardiac glycosides, as well as mammalian target of rapamycin inhibitors, which downregulate the SASP^30^. Also, elimination of senescent cells using engineered CAR T cells leads to improved tumor control in combination with MEK inhibitors and palbociclib, an inhibitor of CDK4 and CDK6^32^. Senescent cells express immunosuppressive ligands, including PD-L1 and CD80, in the context of aging and cancer^33–35^. Interfering with PD-L1 or CD80 signaling promotes the immune clearance of senescent cells, resulting in beneficial effects on healthspan^35^ and on cancer control when combined with chemotherapy^34^. Whether PD-L2 has a role in the senescent phenotype and whether this feature can be exploited in clinical therapies is unknown. In this study, we show the importance of PD-L2 upregulation in the persistence of therapy-induced senescent cells and, thereby, in their ability to modulate tumor immunosurveillance across several mouse models of cancer.

## Results

### Senescent cancer cells overexpress PD-L2

**Fig. 1:**
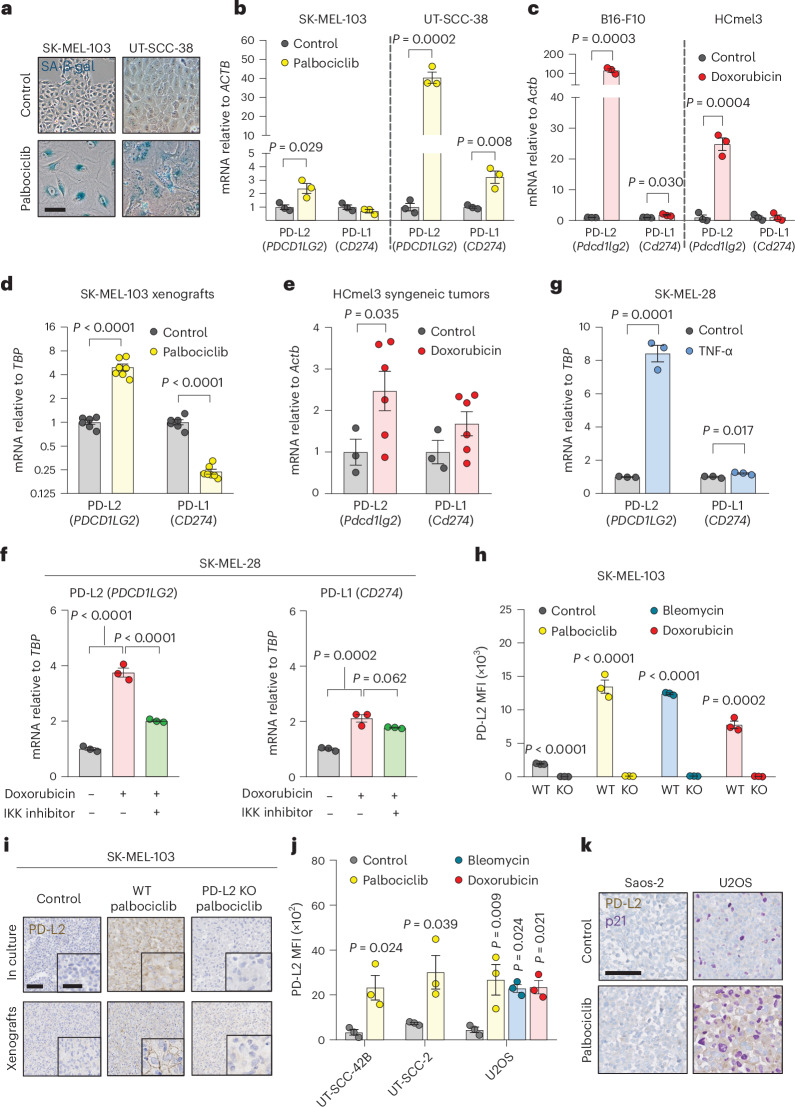
PD-L2 is upregulated in human and murine cancer cell lines on induction of cellular senescence. **a**,**b**, Representative images of SA-β-gal staining (**a**, scale bar, 50 μm) and PD-L1 and PD-L2 mRNA expression in human cancer cell lines treated with palbociclib (**b**, *n* = 3 experiments). **c**, PD-L1 and PD-L2 mRNA expression in murine cancer cell lines after treatment with doxorubicin (*n* = 3 experiments). **d**, PD-L1 and PD-L2 mRNA expression in SK-MEL-103 xenograft tumors in nude mice, treated with 100 mg kg^−1^ oral palbociclib for 10 days and euthanized after the treatment. Control (*n* = 6 tumors); palbociclib-treated tumors (*n* = 7). **e**, PD-L1 and PD-L2 mRNA expression in HCmel3 tumors in C57BL/6 mice treated with 5 mg kg^−1^ doxorubicin (days 7, 10 and 17), analyzed after day 19. Control (n = 3 tumors); doxorubicin-treated tumors (*n* = 6). **f**, PD-L1 and PD-L2 mRNA expression in SK-MEL-28 cells treated with doxorubicin and then with the IKK inhibitor BAY 11-7082 (3 μM, 24 h, *n* = 3 experiments). **g**, PD-L1 and PD-L2 mRNA expression in SK-MEL-28 cells treated with TNF-α (100 ng ml^−1^, 3 days, *n* = 3 experiments). **h**, Quantification of PD-L2 protein levels using flow cytometry on the generation of a PD-L2 KO SK-MEL-103 cell line, under control and senescent conditions (*n* = 3 experiments). **i**, PD-L2 staining of WT and PD-L2 KO SK-MEL-103 cells in culture (as cell pellets) and as xenograft tumors, untreated and treated with palbociclib. Scale bar, 100 μm. Insets: high-magnification images. Scale bar, 50 μm. Representative images of *n* = 3 experiments. **j**, PD-L2 protein levels measured using flow cytometry after induction of senescence with palbociclib, bleomycin or doxorubicin in different cancer cell lines on day 7 after induction. MFI, mean fluorescence intensity (*n* = 3 experiments). Data are presented as the mean ± s.e.m. Two-sided *t*-tests or a one-way analysis of variance (ANOVA) with Tukey post-hoc test were used. **k**, Representative images of cell pellets stained for p21 and PD-L2 in Saos-2 and U2OS. Double staining was performed once. Scale bar, 100 μm. For all the experiments in culture, senescence was induced with 200 nM doxorubicin for 48 h, 5 µM palbociclib for 7 days or 12 mU bleomycin for 48 h. Senescence was evaluated at day 7. Source data

**Extended Data Fig. 1:**
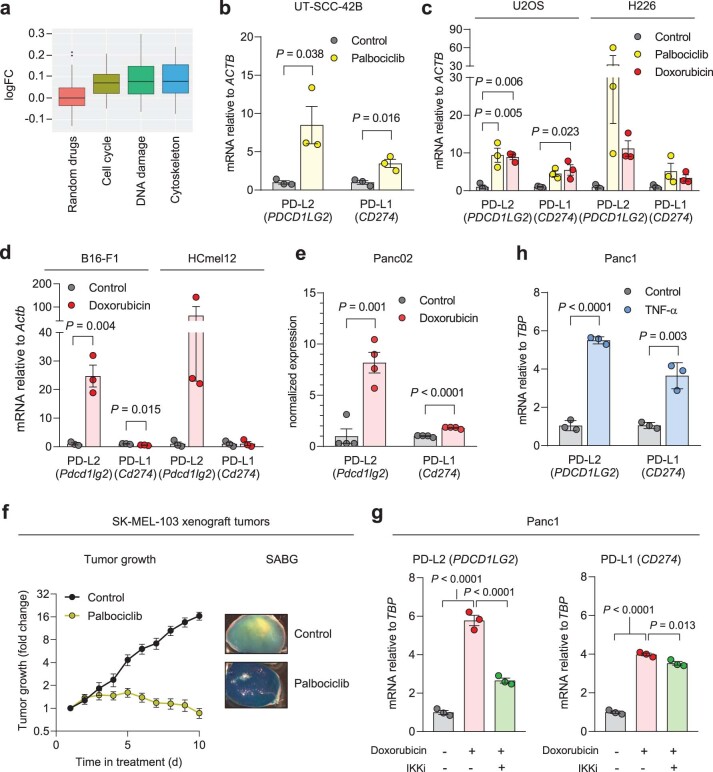
PD-L2 is upregulated in human and murine senescent cancer cells. (a) Drug class enrichment analysis for human *PDCD1LG2* (PD-L2). *P* = 0.0013 for cell cycle, *P* = 8.3 · 10^-^^06^ for DNA damage, *P* = 0.0003 for cytoskeleton. (b) PD-L1/2 mRNA expression in human cancer cell lines treated with palbociclib. (c) PD-L1/2 mRNA expression in human cancer cell lines after treatment with doxorubicin or palbociclib. (d) PD-L1/2 mRNA expression in murine cancer cell lines treated with doxorubicin. N = 3 experiments for (b-d). (e) Normalized PD-L1/2 mRNA expression measured by RNA sequencing. N = 4 biological replicates. (f) Growth curve and representative whole mount beta-galactosidase stainings of SK-MEL-103 xenografts in nude mice, untreated or treated with 100 mg/kg oral palbociclib, daily, once tumors reached 150 mm^3^ (day 7-10). (g) PD-L1/2 mRNA expression in SK-MEL-103 cells treated with doxorubicin and the IKK inhibitor BAY 11-7082 (3 µM, 24 h). N = 11 control mice and 14 palbociclib-treated mice. (h) PD-L1/2 mRNA expression in SK-MEL-103 cells treated with TNF-α (100 ng/ml, 3 days). N = 3 experiments for (g-h). Data are presented as mean ± SEM. Two-sided t-tests or 1 way ANOVA with Tukey post-test were applied. For all the experiments in culture, senescence was induced with 200 nM doxorubicin for 48 h, 5 µM palbociclib for 7 days or 12 mU bleomycin for 48 h. Senescence was evaluated at day 7. Source data

To gain an insight into the immunomodulatory potential of senescent cells, we performed an unbiased proteomic screen of proteins enriched in the plasma membrane of senescent and non-senescent human SK-MEL-103 melanoma cells, using doxorubicin or palbociclib to trigger TIS^36^. Interestingly, among the upregulated plasma membrane proteins in both TIS conditions (doxorubicin and palbociclib) was PD-L2 (Supplementary Table 1). Moreover, drug class enrichment analysis of datasets from the LINCS L1000 project^37^ revealed that PD-L2 is upregulated by known inducers of cellular senescence, such as DNA damage agents and cell cycle inhibitors (Extended Data Fig. 1a). Indeed, PD-L2 transcript levels were upregulated in TIS in cancer cell lines of several origins, namely, human melanoma (SK-MEL-103), mouse melanoma (B16-F1, B16-F10, HCmel3, HCmel12), mouse pancreatic adenocarcinoma (Panc02), human lung squamous cell carcinoma (H226), human head and neck squamous cell carcinoma (UT-SCC-2, UT-SCC-38, UT-SCC-42B), human pancreatic adenocarcinoma (Panc1), and human osteosarcoma (U2OS) (Fig. 1a–c and Extended Data Fig. 1b–g). In contrast, increases in PD-L1 transcript levels were modest or absent. PD-L2 expression was also elevated in vivo after the induction of TIS in human xenografts and syngeneic mouse tumors (Fig. 1d,e and Extended Data Fig. 1f). A conserved feature of cellular senescence is the constitutive hyperactivation of the transcription factor NF-kB, which is a key driver of the SASP^38,39^. Interestingly, blunting NF-kB signaling by inhibition of its upstream activator IκB kinase (IKK) in human melanoma (SK-MEL-28) and pancreatic adenocarcinoma (Panc1) on doxorubicin treatment of senescent cells resulted in a marked decrease of PD-L2 mRNA levels while PD-L1 expression was largely unaffected (Fig. 1f and Extended Data Fig. 1g). Incubation of these cancer cell lines with tumor necrosis factor-α (TNF-α), which activates NF-κB, also induced PD-L2 expression to a larger extent than PD-L1 (Fig. 1g and Extended Data Fig. 1h). Together, these data show that chemotherapy-induced NF-κB signaling is a strong inducer of PD-L2, but not of PD-L1, mRNA levels.

**Extended Data Fig. 2:**
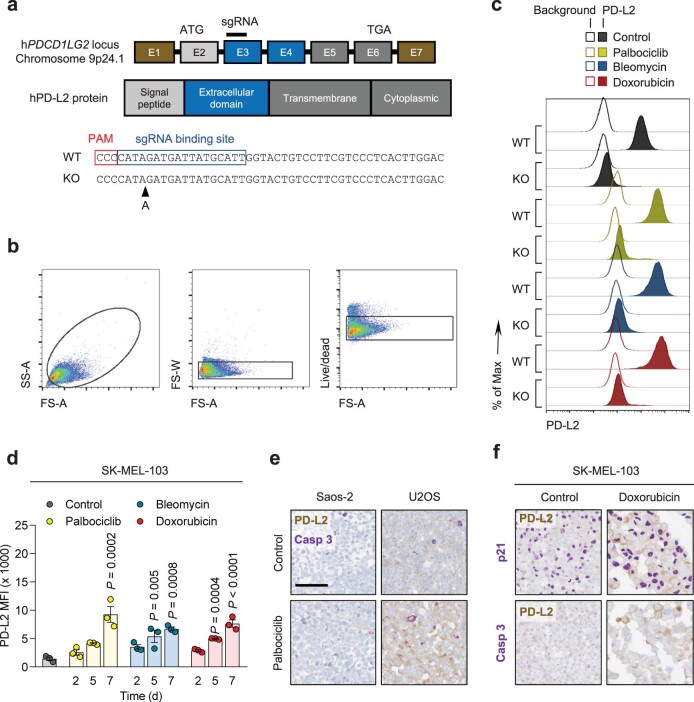
PD-L2 protein levels are upregulated in human and murine senescent cells. (a) CRISPR-Cas9 genome editing of the human *PDCD1LG2* locus, specifying the sgRNA binding site in exon 3. The sequence corresponds to the single clone of edited SK-MEL-103 cells used in the experiments labelled as PD-L2-KO SK-MEL-103. (b) Gating strategy for definition of PD-L2 positive populations in culture and (c) representative example (1 out of n = 3) of histogram for PD-L2 protein levels upon generation of a PD-L2-KO SK-MEL-103 cell line, in control and senescent conditions, measured by flow cytometry. The gating strategy in (b) applies to Figs. 1h, 1j and panel (d) in the present figure. (d) PD-L2 protein levels as measured by flow cytometry upon induction of senescence with palbociclib, bleomycin and doxorubicin in SK-MEL-103 cells. N = 3 independent experiments. Data are presented as mean ± SEM. 1-way ANOVA was applied. (e) Double staining of PD-L2 and active caspase-3 in Saos-2 and U2OS cell pellets, after treatment with palbociclib. (f) Double staining of PD-L2 and p21 (above) and PD-L2 and caspase-3 (below) in SK-MEL-103 cell pellets, after treatment with doxorubicin. Double stainings in (e) and (f) were performed once. Scale bars = 100 μm. For all the experiments in culture, senescence was induced with 200 nM doxorubicin for 48 h, 5 μM palbociclib for 7 days (unless otherwise indicated) or 12 mU bleomycin for 48 h. Senescence was evaluated at day 7 unless otherwise indicated. Source data

Based on these findings, we next assessed the cell surface levels of PD-L2 in TIS. Flow cytometry and immunohistochemistry (IHC)-based analyses revealed elevated PD-L2 in SK-MEL-103 cells, both in culture and in xenograft models, on TIS (Fig. 1h,i). As expected, PD-L2 was not present in a PD-L2 knockout (KO) SK-MEL-103 clonal cell line generated by CRISPR–Cas9 after senescence induction (Fig. 1h,i and Extended Data Fig. 2a–c). Upregulation of PD-L2 was detected using flow cytometry in cell lines of different cancer cell types that underwent TIS (U2O2, and human head and neck carcinoma UT-SCC-2 and UT-SCC-42B) (Fig. 1j) and occurred gradually during the development of TIS over 7 days (Extended Data Fig. 2d). Interestingly, osteosarcoma Saos-2 cells, which are unable to undergo senescence (because of the absence of RB1 (ref. ^40^)), did not upregulate PD-L2 in response to palbociclib, in contrast to TIS-competent osteosarcoma U2OS cells (Fig. 1k). Costaining of PD-L2 with p21 confirmed that PD-L2 was expressed in U2OS and SK-MEL-103 senescent cells rather than in cells resistant to TIS; none of the treatments used to induce TIS triggered any noticeable level of apoptosis (Fig. 1k and Extended Data Fig. 2e,f). Together, these data indicated that the upregulation of PD-L2 was a common feature of the program elicited by TIS and resulted in elevated plasma membrane PD-L2 levels.

### PD-L2 KO tumors are highly susceptible to chemotherapy

**Fig. 2:**
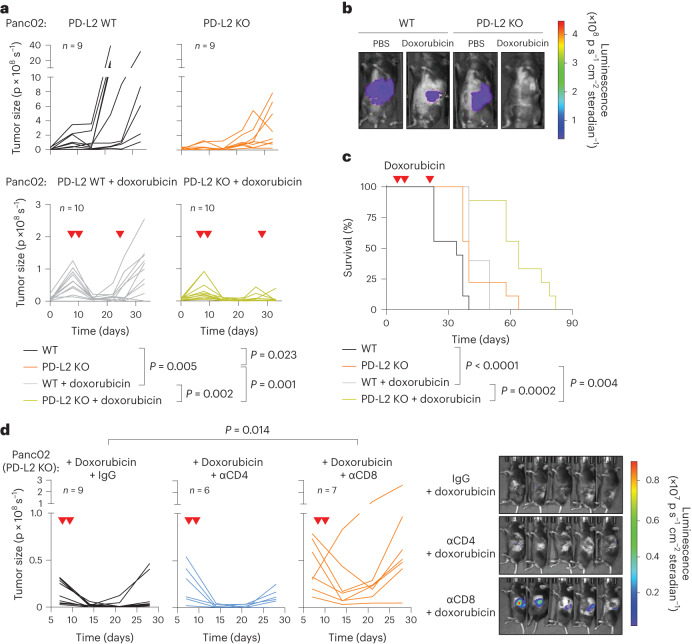
A combination of PD-L2 ablation and chemotherapy results in CD8 T cell-dependent tumor remission. **a**, Quantification of tumor growth for PD-L2 WT and KO Panc02 orthotopic tumors, untreated or treated with doxorubicin (4 mg kg^−1^) at days 7, 10 and 24. **b**, Representative images. PBS-injected groups (*n* = 9 mice); doxorubicin-treated groups (*n* = 10). **c**, Survival curve for the mice from **a**. **d**, Quantification and representative images of tumor growth for PD-L2 KO Panc02 tumors after doxorubicin treatment (4 mg kg^−1^, days 7 and 10) and repeated injections with IgG isotype control, anti-CD4 or anti-CD8 blocking antibodies (100 μg per injection) from day 3 after tumor cell injection and repeated every 3 days. Inverted red triangles indicate day of doxorubicin treatment. Luminescence units are photons (p) s^−1^ in the graphs and p s cm^−^^2^ steradian in the images. A two-way ANOVA and one-way ANOVA with Tukey post-hoc test were used. Source data

**Extended Data Fig. 3:**
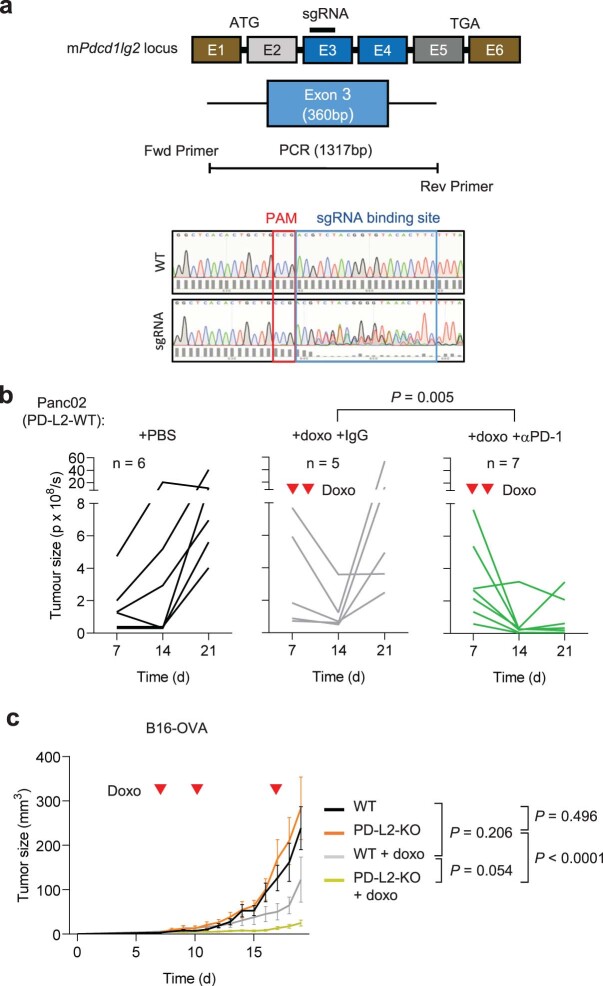
A combination of PD-L2 ablation and chemotherapy results in tumor remission. (a) CRISPR-Cas9 genome editing of the murine *Pdcd1lg2* locus, specifying the sgRNA binding site in exon 3, that generated a bulk population of edited Panc02 cells. This bulk population was used in all the experiments labelled as PD-L2-KO Panc02. (b) Quantification of tumor growth for PD-L2-WT tumors, untreated or treated with doxorubicin on days 7 and 10, including an additional group treated with anti-PD-1 depleting antibody (200 µg), or the same dose of IgG isotype control, from day 3 after tumor cell injection, twice a week. N = 6 for PBS-injected mice, n = 5 for doxo-treated, n = 7 for doxo + anti-PD-1. (c) Growth curve of WT and bulk PD-L2-KO B16-OVA tumors in C57BL/6 mice, untreated or treated with doxorubicin on days 7, 10 and 17 after subcutaneous injection of cells. B16-OVA-WT n = 5 tumors, B16-OVA-KO n = 6, B16-OVA-WT + doxo n = 11, B16-OVA-KO + doxo n = 11. 2-way ANOVA. Source data

The consistent upregulation of PD-L2 in therapy-induced senescent cells prompted us to study its relevance in the context of cancer therapy. For this, we generated murine PD-L2 KO Panc02 cells using CRISPR–Cas9 (Extended Data Fig. 3a) and we injected wild-type (WT) and PD-L2 KO cells orthotopically into the pancreas of immunocompetent C57BL/6 mice, which were subsequently treated with genotoxic chemotherapy (doxorubicin). Interestingly, chemotherapy controlled the growth of PD-L2 KO tumors significantly better than their WT counterparts (Fig. 2a,b). Thus, mice bearing PD-L2 KO tumors and treated with doxorubicin lived significantly longer than mice with treated WT tumors or untreated PD-L2 KO tumors (Fig. 2c). A combined treatment of doxorubicin and anti-PD-1 blocking antibody also provided additional protection compared to doxorubicin alone in WT tumors (Extended Data Fig. 3b). To extend our observations to a different tumor model, we injected WT and PD-L2 KO B16-OVA melanoma cells orthotopically into C57BL/6 mice. Again, we observed a profound reduction in tumor growth rate in PD-L2 KO tumors treated with chemotherapy (Extended Data Fig. 3c). In contrast, WT tumors had a partial and transient reduction in tumor growth in response to chemotherapy (Extended Data Fig. 3c). Collectively, these data indicate that the expression of PD-L2 by cancer cells limits the efficacy of chemotherapy.

**Extended Data Fig. 4:**
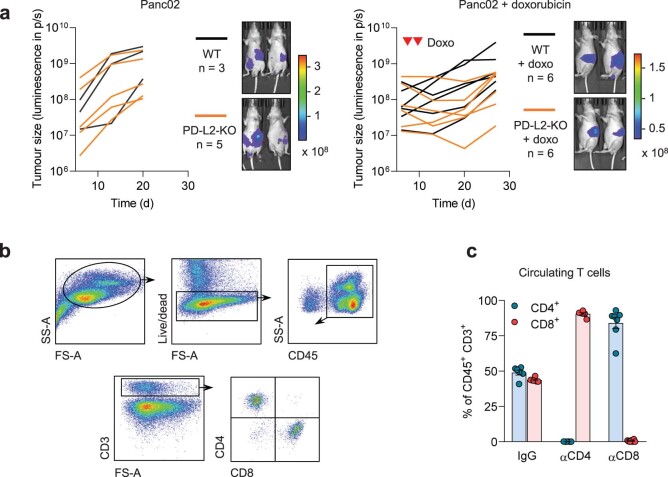
A functional adaptive immune system is required for clearance of PD-L2-KO tumors. (a) Quantification and representative images of Panc02 WT and PD-L2-KO tumors in nude mice, untreated and treated with doxorubicin on days 7 and 10. Luminescence units are photon/sec/cm^2^/stereoradian in the images. N = 3 for PD-L2-WT + PBS mice, n = 5 for PD-L2-KO + PBS, n = 6 for both doxo-treated groups. (b) Gating strategy to define circulating CD4^+^ and CD8^+^ T cell populations. (c) Percentage of CD4^+^ and CD8^+^ T cells among total CD45^+^ CD3^+^ cells in blood of mice after treatment with blocking anti-CD4 and anti-CD8 antibodies. N = 6-7 mice. Data are presented as mean ± SEM. Source data

We next analyzed whether a functional adaptive immune response was required for the control of PD-L2 KO tumors. For this, we injected WT and PD-L2 KO Panc02 cells into the pancreas of athymic nude mice (lacking T cells) and quantified tumor growth over time. Neither in the absence of chemotherapy nor after the administration of doxorubicin (Extended Data Fig. 4a) did tumors show any significant difference in growth rate across experimental groups, suggesting that the adaptive immune system was responsible for the phenotype associated with PD-L2 KO tumors. To elucidate which major T cell subset is essential for tumor regression, we depleted CD4^+^ or CD8^+^ T cells from animals bearing PD-L2 KO pancreatic tumors, which were subsequently treated with doxorubicin (Extended Data Fig. 4b,c). Mice lacking CD8^+^ T cells were unable to control PD-L2 KO tumors after chemotherapy, while control animals or mice treated with anti-CD4-depleting antibodies had robust suppression of tumor growth (Fig. 2d). These results indicated that CD8^+^ T cells were responsible for the efficient removal of PD-L2 KO tumors on chemotherapy.

### PD-L2 suppression impairs the recruitment of myeloid cells after therapy

**Fig. 3:**
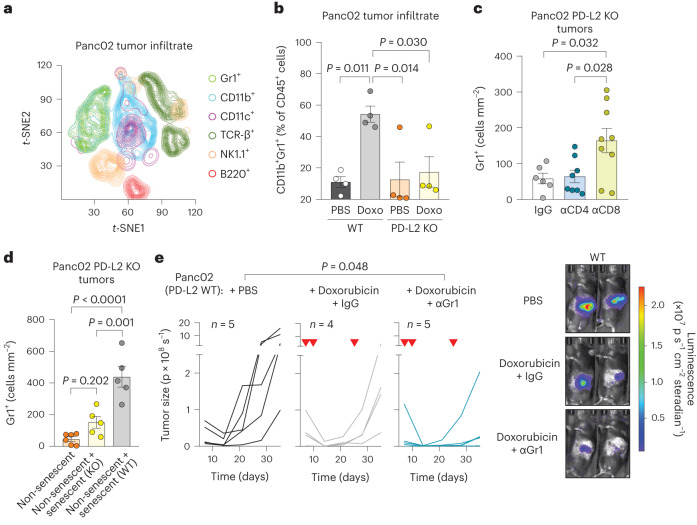
Recruitment of tumor-promoting myeloid cells is prevented in PD-L2 KO after doxorubicin treatment. **a**, *t*-distributed stochastic neighbor embedding (*t*-SNE) plot including the different tumor-infiltrating immune subpopulations detected using mass cytometry. The plot shows the pooled data for a total of 16 mice corresponding to four experimental groups (*n* = 4 mice for WT and PD-L2 KO, untreated or treated with doxorubicin). Doxorubicin (4 mg kg^−1^) treatment was administered on days 7 and 10, and samples were obtained on day 12. **b**, Quantification of the percentage of CD11b^+^Gr1^+^ cells, relative to total CD45^+^ cells, measured using mass cytometry. *n* = 4 tumors for each experimental group. Doxo, doxorubicin. **c**, Quantification of Gr1^+^ cells in sections of PD-L2 KO tumors treated with doxorubicin on days 7 and 10, subject to depletion of CD4^+^ (*n* = 8 mice) or CD8^+^ (*n* = 9) T cells (100 μg per injection, every 3 days from day 3) or the same dose of IgG (*n* = 6) isotype control, and collected on day 28. **d**, Quantification of Gr1^+^ cells in sections of tumors generated by the coinjection of non-senescent (*n* = 6 mice) PD-L2 KO Panc02 cells in combination with senescent Panc02 cells, either WT (*n* = 5) or PD-L2 KO (*n* = 5), evaluated 6 days after tumor cell injection. **e**, Representative quantification and images of tumor growth for PD-L2 WT tumors, untreated or treated with doxorubicin (4 mg kg^−1^) on days 7, 10 and 24, including an additional group treated with anti-Gr1 blocking antibody (200 μg per injection, started on day 3 and continued twice weekly) or the same dose of IgG isotype control (*n* = 4,5 mice per group). Inverted red triangles indicate day of doxorubicin treatment. Luminescence units are p s^−1^ in the graphs and p s cm^−^^2^steradian in the images. Data are presented as mean ± s.e.m. A one-way ANOVA with Tukey post-hoc test was used. Source data

**Extended Data Fig. 5:**
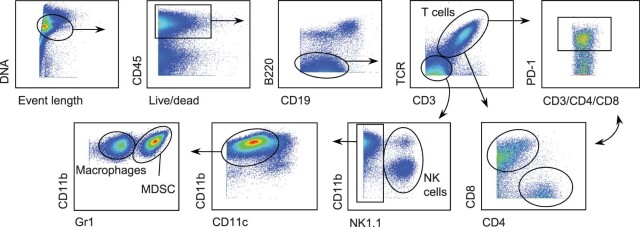
Gating strategy for definition of immune cell populations by mass cytometry. Definition of T cells (including CD4^+^, CD8^+^ and, in each case, PD-1^+^ cells), macrophages, NK cells and myeloid-derived suppressor cells shown in Fig. 3a-b and Extended Data Fig. 6a-b.

**Extended Data Fig. 6:**
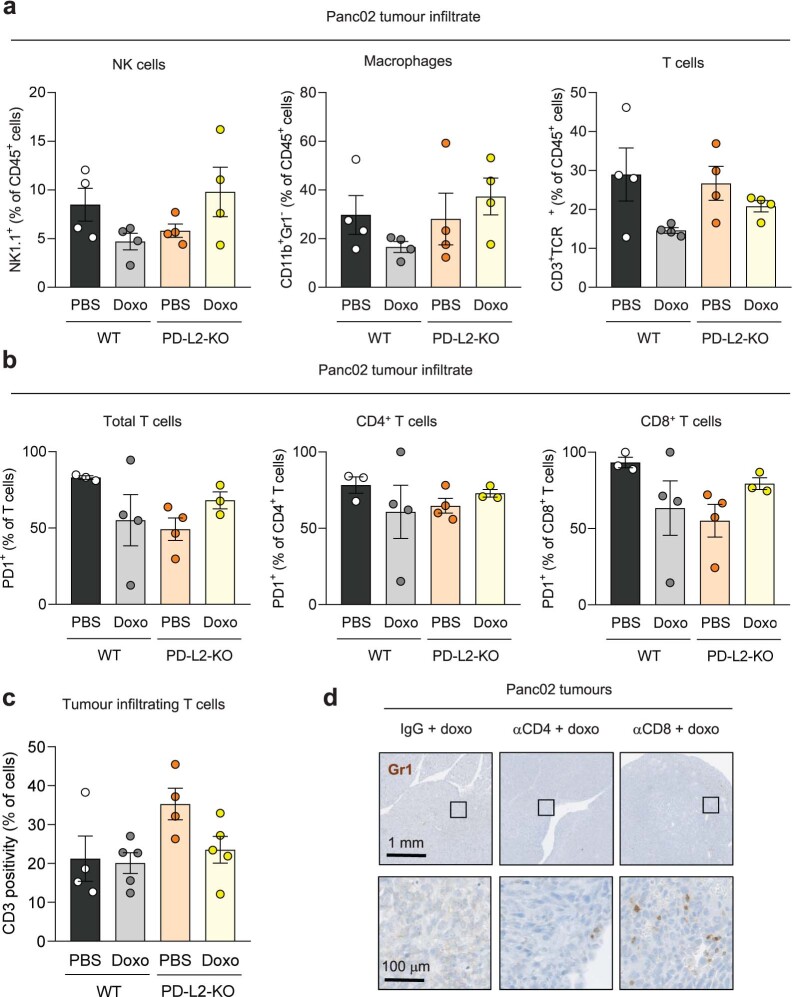
Recruitment of tumor-promoting myeloid cells is prevented in PD-L2-KO tumors upon doxorubicin treatment. (a) Percentage of NK cells (NK1.1^+^), macrophages (CD11b^+^ Gr1^-^) and lymphocytes (CD3^+^ TCRb^+^) relative to total CD45^+^ cells, in WT and PD-L2-KO tumors untreated or treated with doxorubicin at days 7 and 10, analysed by mass cytometry. N = 4 mice for all conditions. (b) Percentage of PD-1^+^ cells among subsets of infiltrating T cells, analysed by mass cytometry. N = 3 mice for WT + PBS and PD-L2-KO + doxo, n = 4 for WT + doxo and PD-L2-KO + PBS (c) Quantification of tumor infiltrating CD3^+^ lymphocytes in WT and PD-L2-KO tumors, untreated or treated with doxorubicin, analysed by immunohistochemistry. N = 4 for PBS-treated groups and n = 5 for doxo-treated groups. None of the changes were significant (1 way ANOVA, Tukey post-test). Data are presented as mean ± SEM. (d) Representative Gr1 stainings in sections of PD-L2-KO tumors treated with doxorubicin and subject to depletion of CD4^+^ (n = 8) or CD8^+^ (n = 9) T cells, as well as IgG-treated controls (n = 6) from Fig. 3c. Source data

To better understand the mechanisms underlying the enhanced efficiency of chemotherapy in PD-L2 KO tumors, we performed a comprehensive analysis of the immune infiltrate in Panc02 PD-L2 WT and KO orthotopic tumors. We quantified a panel of different immune populations using mass cytometry 5 days after the start of doxorubicin treatment (Fig. 3a and Extended Data Fig. 5). We detected a substantial recruitment of CD11b^+^Gr1^+^ myeloid cells in PD-L2 WT tumors after treatment with doxorubicin (Fig. 3b). This was in sharp contrast to PD-L2 KO tumors, which were unable to recruit these myeloid cells (Fig. 3b). No significant variations were observed across experimental conditions for other immune populations, including T cells, macrophages or natural killer (NK) cells (Extended Data Fig. 6a,c). Most of the infiltrating T cells, either CD4 or CD8, expressed PD-1 regardless of treatment and PD-L2 status (Extended Data Fig. 6b). Interestingly, we also observed that depletion of CD8^+^ T cells resulted in the accumulation of Gr1^+^ cells in chemotherapy-treated PD-L2 KO tumors (Fig. 3c and Extended Data Fig. 6d). This suggests that tumor senescent cells lacking PD-L2 were eliminated by CD8^+^ T cells and failure to eliminate them resulted in the recruitment of Gr1^+^ cells. To further define the role of senescent cells in the recruitment of Gr1^+^ cells, we coinjected non-senescent PD-L2 KO Panc02 orthotopically, together with WT or PD-L2 KO cells rendered senescent in culture. Six days after tumor implantation, Gr1^+^ cells were more abundant in those tumors into which WT senescent cells were coinjected, unlike those initially containing PD-L2 KO senescent cells or only PD-L2 KO non-senescent growing cells (Fig. 3d). This also demonstrated that intratumoral senescent cells, in the absence of systemic chemotherapeutic treatment, were directly responsible for the recruitment of myeloid cells. To evaluate the impact of Gr1^+^ myeloid cells in orthotopic Panc02 tumors, we depleted Gr1^+^ cells from animals bearing WT tumors and treated with doxorubicin, observing an improvement in tumor control (Fig. 3e). These results indicate that, on chemotherapy, immunosuppressive myeloid cells were recruited by senescent cells in a PD-L2-dependent manner. Conversely, absence of PD-L2 facilitated the elimination of senescent cells by CD8^+^ T cells and this prevented the recruitment of Gr1^+^ immunosuppressive myeloid cells after chemotherapy, further facilitating the action of CD8^+^ T cells on the entire tumor cell population.

### PD-L2 is not required for cellular senescence

**Extended Data Fig. 7:**
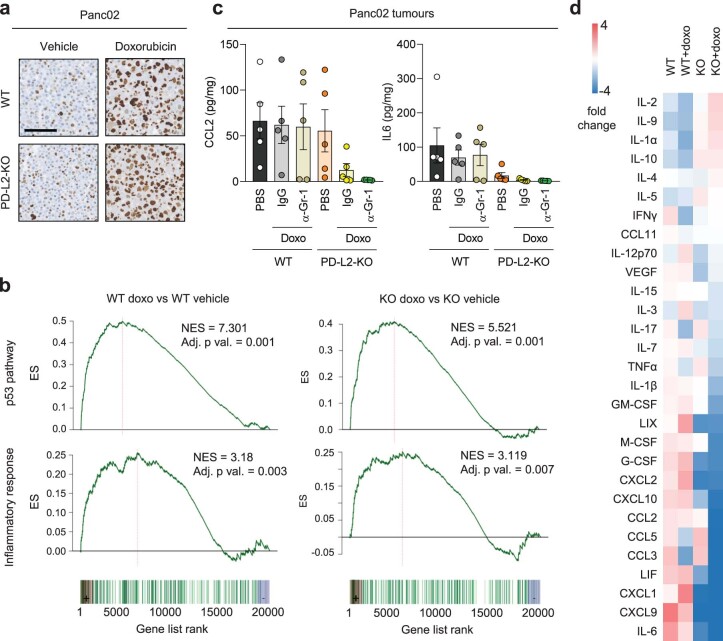
Characterization of senescent cells in culture and in vivo. (a) p21 staining in pellets from Panc02 cells, WT and PD-L2-KO, untreated or treated with doxorubicin (200 nM, 48 h, evaluated at day 7, n = 4 experiments). Scale bar = 100 μm. (b) GSEA plots for representative gene sets associated to cellular senescence in doxorubicin-treated versus untreated WT Panc02 cells, as well as in doxorubicin-treated (as described) versus untreated PD-L2-KO Panc02 cells at day 7 after treatment. DESEq2 was used for differential expression analysis with fold change shrinkage as implemented in the lfcShrink function. Functional enrichment analysis was performed over gene sets defined in the Molecular Signatures Database (MSigDB) hallmark gene set collection. Data from 4 biological replicates. (c) Absolute quantifications of intratumoral levels of CCL2 and interleukin 6 measured by a commercial multiplexed system with beads bound to antibodies, in WT and PD-L2-KO tumors, untreated or treated with doxorubicin (4 mg/kg, days 7, 10 and 24) and anti-Gr1 (200 µg per injection, as described for Fig. 3e), or the same dose of IgG isotype control. N = 5 mice per experimental condition. 1 way ANOVAs with Tukey post-tests were applied. (d) Relative levels of intratumoral cytokines and chemokines in WT and PD-L2-KO tumors, untreated or treated with doxorubicin (4 mg/kg, on days 7, 10 and 24, as described). N = 5 mice per experimental condition. Source data

While PD-L2 modulates the function of immune cells, we wondered if it could also affect the process of cellular senescence. Panc02 PD-L2 WT and KO cells presented a similar senescent morphology and p21 levels on treatment with doxorubicin (Extended Data Fig. 7a). To gain a deeper understanding of potential differences, we obtained their expression profiles by RNA sequencing (RNA-seq) and we found that they were essentially indistinguishable (only ten genes were differentially expressed out of approximately 21,000 detected genes (fold change > 1.5, false discovery rate (FDR) < 0.05)) (Supplementary Table 2). Notably, the highest upregulated mRNA in PD-L2 KO cells was the CRISPR-inactivated PD-L2 encoding gene (*PDCD1LG2*). This upregulation revealed a layer of regulation of PD-L2 consistent with a negative feedback loop between functional PD-L2 protein and expression of PD-L2-coding mRNA. Relevant for our current work, the enrichment of signatures of p53 activation and inflammation were evident and similar in WT and KO doxorubicin-treated Panc02 cells (Extended Data Fig. 7b). We conclude that PD-L2 is not relevant for the induction of senescence in vitro, nor for the inflammatory program of senescent cells.

### PD-L2 determines the persistence of tumor senescent cells

**Fig. 4:**
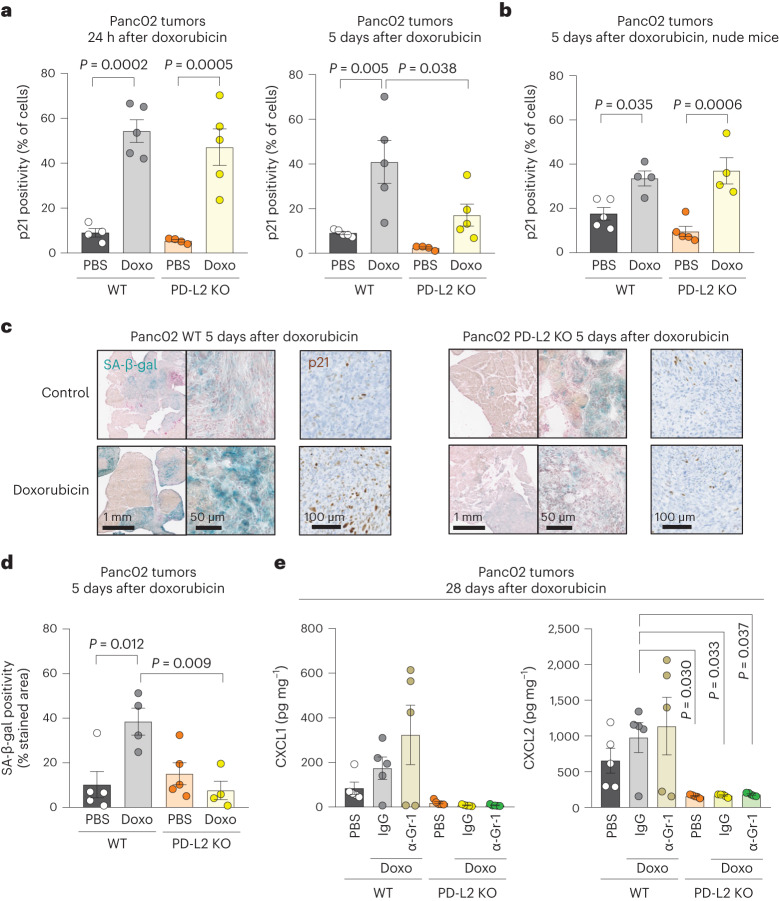
PD-L2 KO Panc02 tumors after chemotherapy present lower levels of cellular senescence markers and intratumoral cytokines and chemokines. **a**, Quantification of p21^+^ cells using IHC in PD-L2 WT and KO Panc02 tumors treated with one dose of doxorubicin (4 mg kg^−1^) on day 7 and analyzed 24 h after (left), or treated twice with the same dose of doxorubicin at days 7 and 10, and analyzed at day 12 (that is, 5 days after the first dose of doxorubicin). PBS-injected groups (*n* = 4 mice); doxorubicin-treated group for the 24 h experiment (*n* = 5 mice); all groups at 5 days (*n* = 5 mice) except for PD-L2 KO + PBS (*n* = 4). **b**, Same experimental design as in **a** (right) in nude mice. Doxorubicin-treated groups (*n* = 4 mice), PBS-injected group (*n* = 5 mice). **c**, Representative staining of p21 and SA-β-gal in Panc02 tumors corresponding to the experiment in **a**. **d**, Quantification of the SA-β-gal of **c**. Doxorubicin-treated groups (*n* = 4 mice); PBS-injected group (*n* = 5 mice). **e**, Intratumoral levels of CXCL1 and CXCL2 in PD-L2 WT and KO tumors, treated with doxorubicin as in **a**. Treatment with anti-Gr1 (200 μg per injection) or the same dose of IgG isotype control was started on day 3 and continued twice a week, as described for Fig. 3e. Mice per experimental group in all assays (*n* = 5). Data are presented as the mean ± s.e.m. A one-way ANOVA with Tukey post-hoc test was used. Source data

To evaluate the levels of senescence in orthotopic pancreatic tumors, we initially quantified p21^+^ cells using IHC. In agreement with our in vitro data, the induction of p21 at a short time after doxorubicin (24 h) was similar in PD-L2 WT and KO tumors (Fig. 4a, left). Interestingly, however, 5 days after chemotherapy, the levels of p21^+^ were clearly decreased in PD-L2 KO tumors but not in PD-L2 WT tumors (Fig. 4a). To assess the role of the adaptive immune system in the clearance of PD-L2 KO senescent cells, we performed the same experiment in athymic nude mice. In this case, PD-L2 KO p21^+^ cells persisted to the same extent as PD-L2 WT p21^+^ cells (Fig. 4b). The differential persistence of PD-L2 WT and KO senescent cells was also confirmed by senescence-associated β-galactosidase (SA-β-gal) staining and quantification (Fig. 4c,d). We conclude that the absence of PD-L2 impaired the persistence of intratumoral senescent cells after therapy.

Senescent cells contribute to the generation of an immunosuppressive microenvironment by recruiting MDSCs into the tumor^26–29^. Several cytokines and chemokines present in the SASP have been implicated in MDSC recruitment, including interleukin-6 (IL-6), CCL2, CXCL1 and CXCL2 (ref. ^28^). We quantified the intratumoral levels of these chemokines and cytokines in our Panc02 model and observed that they were reduced in PD-L2 KO tumors after chemotherapy, this was particularly the case for CXCL1, CXCL2 and IL-6 (Fig. 4e and Extended Data Fig. 7c); several other cytokines and chemokines also showed a similar trend (Extended Data Fig. 7d). While intratumoral myeloid cells could contribute to the production of these cytokines and chemokines, depletion of Gr1^+^ cells did not result in decreased intratumoral levels of any of the most relevant factors in PD-L2 WT tumors (Fig. 4e and Extended Data Fig. 7c). Our results support a model in which, after therapy, senescent cells induce intratumoral immunosuppression by expressing PD-L2 and by recruiting suppressive myeloid cells.

### Combinatorial therapy with blocking anti-PD-L2 antibodies

**Fig. 5:**
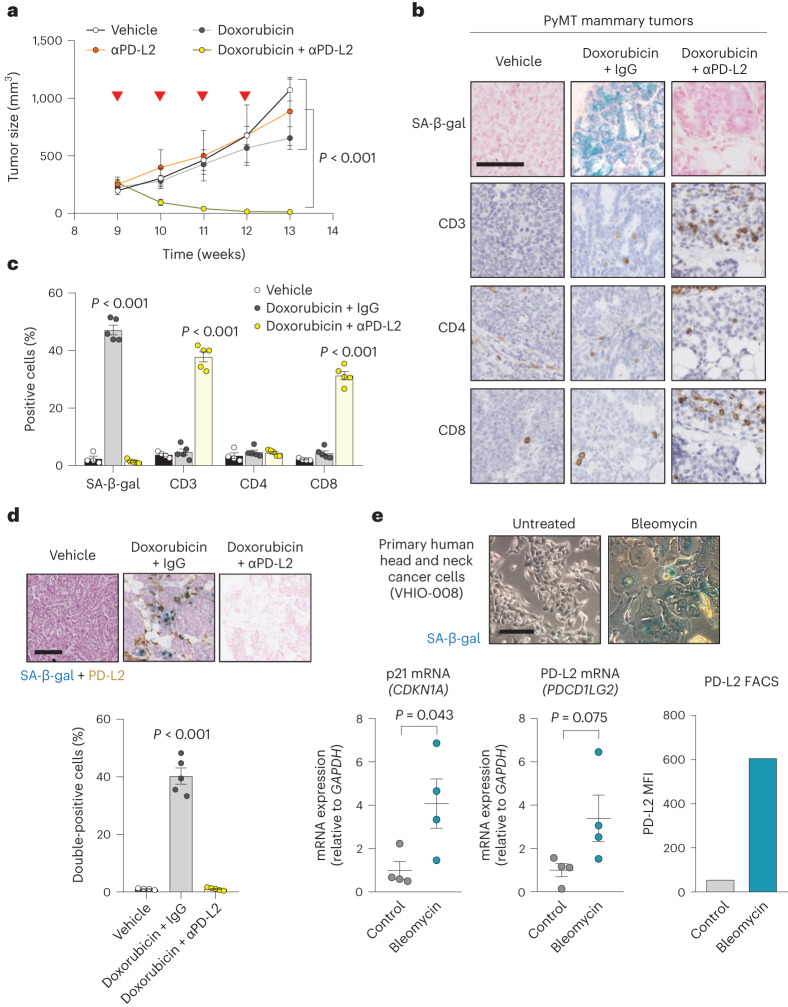
Combining chemotherapy and PD-L2 blockade holds translational potential. **a**, Tumor growth in PyMT mice treated weekly (from week 9 until week 12) with αPD-L2 alone (TY25, 10 mg kg^−1^) or the same dose of isotype control every 3 days, doxorubicin alone or a combination of both as indicated. αPD-L2 monotherapy (*n* = 3 mice); PBS and doxorubicin + IgG groups (*n* = 4); doxorubicin + αPD-L2 (*n* = 5). Two-way ANOVA. Inverted red triangles indicate day of doxorubicin treatment. **b**,**c**, SA-β-gal, CD3, CD4 and CD8 staining of untreated tumors and tumors treated with doxorubicin alone or in combination with αPD-L2 blocking antibody (TY25, 10 mg kg^−1^) or the same dose of isotype control (**b**), as in **a**, and analyzed at week 13 (**c**). Vehicle-treated group (*n* = 4); doxorubicin-treated groups (*n* = 5). Representative images are shown. Statistical significance shown versus the rest of the experimental conditions. One-way ANOVA with Tukey post-hoc test. Scale bar, 100 μm. **d**, SA-β-gal and PD-L2 costaining in tumor samples from PyMT mice treated weekly with doxorubicin (4 mg kg^−1^) at postnatal weeks 9–12. The indicated groups were treated with αPD-L2 (TY25, 10 mg kg^−1^), or the same dose of IgG isotype control, every 3 days. Representative images and quantification are shown. Samples were obtained at postnatal week 13 (that is, 7 days after the last doxorubicin dose). Vehicle-treated group (*n* = 4 mice); doxorubicin-treated groups (*n* = 5 mice). A one-way ANOVA was used for significant changes versus the rest of the conditions. Scale bar, 100 μm. **e**, SA-β-gal staining, p21 mRNA levels, PD-L2 mRNA levels and PD-L2 protein levels measured using fluorescence-activated cell sorting (FACS), with and without bleomycin treatment, in human head and neck cancer primary culture from patient VHIO-008. Gene expression (*n* = 4 independent replicates); FACS (*n* = 1). Scale bar, 50 μm. Two-sided *t*-tests were used. Source data

**Extended Data Fig. 8:**
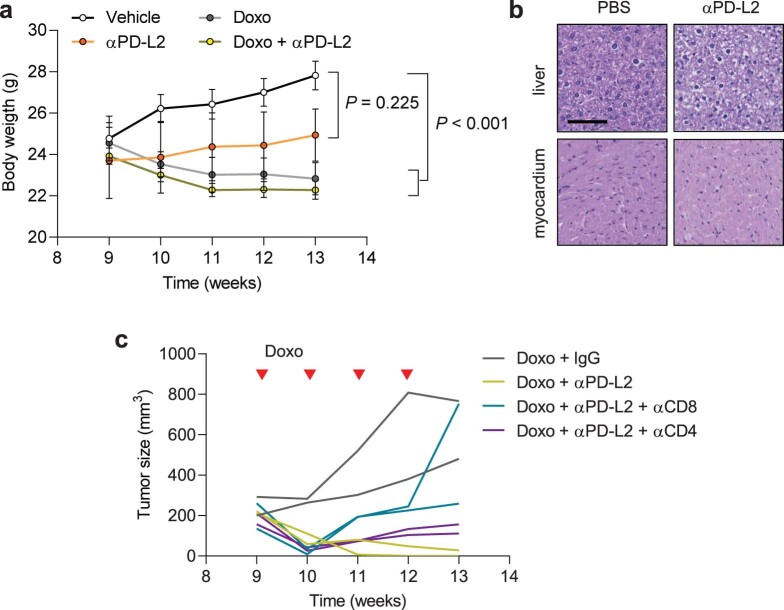
PD-L2 blockade eliminates mammary tumors after chemotherapy. (a) Body weight of PyMT mice during doxorubicin and anti-PD-L2 treatments, corresponding to Fig. 5a. 2-way ANOVA. N = 3 mice for anti-PD-L2 monotherapy, n = 4 for PBS and doxo + IgG groups, n = 5 for doxo + anti-PD-L2. (b) Representative hematoxilin/eosin staining of liver and myocardium of animals treated with anti-PD-L2 (10 mg/kg) (n = 2 mice) or vehicle (PBS, n = 1) every three days for four weeks. Scale bar = 100 μm. (c) Individual replicates (n = 2 mice) of tumor growth curves in PyMT mice treated with doxorubicin and anti-PD-L2, together with blocking antibodies against CD4 (10 mg/kg) and CD8 (10 mg/kg), or the same dose of IgG isotype control, every three days for four weeks. Source data

Given our previous results, we explored the role of PD-L2 activity in additional cancer models. Mouse mammary tumor virus (MMTV)-PyMT mice develop spontaneous mammary gland tumors, reaching the status of adenoma and carcinoma at 9 and 13 weeks of age, respectively. To determine the role of PD-L2 in this setting, we used a commercially available blocking anti-PD-L2 antibody (αPD-L2, TY25). Treatment with TY25 alone did not have a significant effect on tumor growth; doxorubicin alone had a modest effect in reducing the tumor growth rate (Fig. 5a). Remarkably, the combination of both treatments, TY25 and doxorubicin, resulted in complete tumor regression (Fig. 5a). Histological analysis of the liver and heart from mice treated with anti-PD-L2 antibody did not reveal major morphological changes; the body weight of these mice was only mildly affected (Extended Data Fig. 8a,b). In agreement with the Panc02 orthotopic cancer model, we observed abundant senescent cells (SA-β-gal^+^) in tumors after therapy at 13 weeks of age (Fig. 5b,c). Notably, SA-β-gal^+^ cells were also PD-L2^+^ using IHC, further confirming that PD-L2 is a marker of TIS (Fig. 5d). To characterize the antitumor effect of combined chemotherapy and PD-L2 antibody, we analyzed lymphocytic infiltration using IHC in residual tumors at 13 weeks of age and observed a highly significant increase of CD8^+^ T cells, but not CD4^+^ T cells (Fig. 5b,c). To test the role of CD8^+^ T cells in the remission elicited by anti-PD-L2 treatment, we performed another experiment including anti-CD8-depleting and anti-CD4-depleting antibodies. While targeting CD4^+^ cells had little effect, absence of CD8^+^ cells led to tumor regrowth (Extended Data Fig. 8c). Together, these results extend to mammary tumors the concept that PD-L2 inactivation synergizes with chemotherapy by improving immune surveillance.

### Therapy induces PD-L2 expression in primary human cancer cells

**Extended Data Fig. 9:**
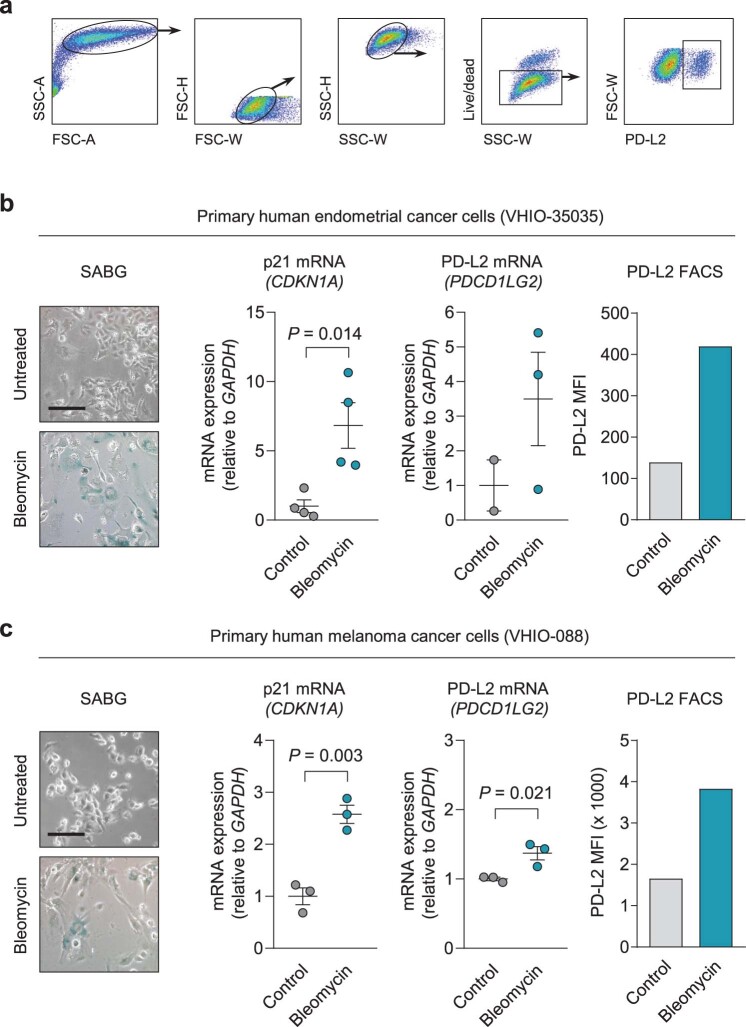
PD-L2 expression and protein levels increase in primary cultures from human patients upon establishment of cellular senescence. (a) Gating strategy for detection of PD-L2^+^ cells in Fig. 5e and in the following panels. (b-c) SABG staining, p21 mRNA levels, PD-L2 mRNA levels, and PD-L2 protein levels measured by FACS with and without bleomycin treatment in (b) human endometrial cancer primary culture from patient VHIO-35035 and (c) human melanoma primary culture from patient VHIO-088. N = 4 independent experiments for gene expression, n = 1 for FACS. Data are presented as mean ± SEM. Two-sided t-tests were applied. Scale bars = 50 μm. Source data

To determine whether PD-L2 is also upregulated in primary cultures from patients with cancer when cells undergo TIS, we established cultures from head and neck cancer (VHIO-008), endometrial cancer (VHIO-35035) and melanoma (VHIO-088) treated with the chemotherapeutic agent bleomycin. Seven days after treatment, all cultures exhibited morphological features of senescence, with SA-β-gal positivity and upregulated p21 (Fig. 5e and Extended Data Fig. 9a–c). Importantly, PD-L2 expression increased concomitantly with the establishment of cellular senescence, as shown by the elevation of PD-L2 mRNA and protein levels, the latter measured using flow cytometry (Fig. 5e and Extended Data Fig. 9a–c). These data support the notion that PD-L2 is a potentially relevant immune checkpoint ligand in patients after therapy.

## Discussion

In this study, we demonstrate that senescence-inducing therapies used in the clinic result in the upregulation of PD-L2 in cancer cells. While the expression of PD-L2 is not necessary for senescence induction or for the secretory phenotype of senescent cells, it critically contributes to the immune evasion of cancer senescent cells in tumors after therapy in vivo. Intratumoral senescent cancer cells do not proliferate but, as we report in this study, favor cancer regrowth after therapy thanks to their immunosuppressive effects. Senescence-mediated immunosuppression is two-pronged, involving (1) the expression of PD-L2 and (2) the secretion of immunosuppressive cytokines and chemokines. Our data indicate that a main function of PD-L2 is to allow immune escape of senescent cells. In turn, senescent cells recruit MDSCs shielding the tumor from immunosurveillance. In agreement with previous studies^26–29^, we report that intratumoral therapy-induced senescent cells release, among multiple cytokines and chemokines, CXCL1, CXCL2 and IL-6, which recruit MDSCs. We show that in the case of tumors lacking PD-L2, senescent cells succumb to CD8^+^ T cells, MDSCs are not recruited and the tumor is vulnerable to immunosurveillance.

The accumulation of senescent cells with aging and in multiple pathological conditions, including cancers, has been partially attributed to defective immunosurveillance by the innate immune system, mainly macrophages and NK cells^41–47^. Additional findings expanded this concept by demonstrating that senescent cells express immune checkpoint ligands such as PD-L1 and CD80 (refs. ^33–35^). It remains to be explored to what extent PD-L2 is also involved in the accumulation of senescent cells associated with aging and aging-associated diseases and whether it has, as suggested by our data, a particularly critical role. The complexity of senescent cells is further enriched by recent reports showing that senescent cells upregulate the major histocompatibility complex class I antigen-presenting machinery^36,48^ and have an altered immunopeptidome that makes them highly immunogenic^36^. The emerging picture is that cellular senescence is under immune surveillance by the innate and adaptive immune system, with the final outcome being dependent on a fine balance between stimulatory and inhibitory signals. Understanding this balance may open therapeutic opportunities to induce immune clearance of senescent cells, as we show in this study in the case of therapy-induced senescent cancer cells.

The concept of immune-mediated senescent cell clearance being synergistic with senescence-inducing therapies has been recently reported in the context of cancer^32^. Specifically, in a model of pulmonary adenocarcinoma, a senescence-inducing therapy followed by the administration of uPAR-specific CAR T cells, which preferentially target senescent cells, extended survival and increased lymphocytic infiltration^32^. Our current findings on the use of anti-PD-L2 antibodies support their potential as a strategy to improve the efficacy of anticancer therapies. It is important to mention that PD-L2 has two known receptors, namely, PD-1 and RGMb, the latter binding PD-L2 and not PD-L1 (ref. ^18^). A very recent report unveiled that RGMb is expressed in CD8^+^ tumor-infiltrating T cells where it acts as an immunosuppressive receptor on binding to PD-L2 (ref. ^19^). The fact that PD-L2 may induce CD8^+^ T cell suppression through two receptors, PD-1 and RGMb, adds further interest on its therapeutic targeting. Future research will determine the extent to which this therapeutic opportunity can be successfully translated to the clinic.

## Methods

### Mouse husbandry

All procedures were approved by the Ethics Committee for Animal Experimentation at the Parc Científic de Barcelona (Spain), the Spanish National Cancer Research Center-Instituto de Salud Carlos III Ethics Committee for Research and Animal Welfare and the Ethics Committee for Animal Experimentation (Ethics Committee for Animal Experimentation) at Vall d’Hebron Institut de Recerca. Male WT mice (C57BL/6) were purchased from Charles River Laboratories. Male nude mice (Hsd:athymic nude-*Foxn1*^*nu*^) were purchased from Envigo. MMTV-PyMT (PyMT) mice on an FVB background were purchased from The Jackson Laboratory. Mice were kept under a 12 h light–dark cycle and were allowed unrestricted access to food and water.

### Mammalian tissue culture

SK-MEL-28 (HTB-72, human melanoma), Panc1 (CRL-1469, human pancreatic adenocarcinoma), U2OS (HTB-96, human osteosarcoma), Saos-2 (HTB-85, human osteosarcoma), HEK 293T (CRL-3216, human embryonic kidney) and H226 (CRL-5826, human squamous cell carcinoma) cells were obtained from ATCC. UT-SCC-38, UT-SCC-42B and UT-SCC-2 cells (human head and neck squamous cell carcinoma) were provided by R. Grenman. SK-MEL-103, B16F1 and B16F10 cells (mouse melanoma) were provided by M. Soengas. B16-OVA: B16 (mouse melanoma) cells expressing ovalbumin (OVA) and Panc02 (mouse pancreatic adenocarcinoma) cells were provided by F. Pietrocola. HCmel3 cells (mouse melanoma) were provided by T. Tüting. None of the cells have been authenticated. Cells were routinely tested for *Mycoplasma* contamination. HcMel3 and Panc02 cells were maintained in Roswell Park Memorial Institute (RPMI) medium (Gibco). The other cell lines were maintained in DMEM (Gibco). All media were supplemented with 10% FCS (Gibco) with 1% penicillin/streptomycin (Gibco). All cell lines were cultured at 37 °C in a humidified atmosphere and 5% CO_2_; procedures were conducted under aseptic conditions in a biosafety cabinet according to standard operating procedures.

### Induction of cellular senescence in tissue culture and treatment with IKK inhibitor and TNF-α

Unless otherwise noted, senescence was induced using 5 μM palbociclib (Pfizer) for 7 days, or 48 h treatment with 200 nM doxorubicin (Sigma-Aldrich) and 12 mU bleomycin (Sigma-Aldrich). Senescence was evaluated at day 7. For IKK inhibition, cells were treated at day 6 with either vehicle (dimethylsulfoxide) or the IKK inhibitor BAY 11-7082 (Selleck Chemicals) at 3 μM for 24 h. Human TNF-α recombinant protein (Thermo Fisher Scientific) was used at 100 ng ml^−1^ for 3 days.

### Gene expression analysis using quantitative PCR

Total RNA from adherent cells or homogenized tissue biopsies was isolated using TRI Reagent (Sigma-Aldrich) according to the manufacturer’s instructions. A total of 3−4 μg of total RNA was reverse-transcribed using the iScript advanced cDNA synthesis kit (Bio-Rad Laboratories). Quantitative PCR (qPCR) of target genes was performed using SYBR Green (Applied Biosystems) and ran on a QuantStudio 6 Flex Real-Time PCR System using the QuantStudio 6 and 7 Flex Real-Time PCR software v.1.0 (Applied Biosystems). Relative gene expression levels were expressed as a function of murine *Actb* or human *TBP* as housekeeping genes, as indicated.

For gene expression determination after IKK inhibitor and TNF-α treatments, RNA was isolated using TRIzol according to the manufacturer’s instructions. Complementary DNA was generated using 1 μg RNA using M-MLV Reverse Transcriptase (Thermo Fisher Scientific) according to the manufacturer’s instructions. qPCR was performed using the TaqMan Fast Advanced Master Mix (Thermo Fisher Scientific) on QuantStudio 7 Pro (Thermo Fisher Scientific). *TBP* was used as the housekeeping gene. In all cases, primers can be found in Supplementary Table 3.

### Generation of cell pellets for IHC

Cells were collected using phosphate buffered saline (PBS) with 10 mM EDTA at 37 °C and centrifuged at 300*g*, overlaid with 10% buffered formalin (Sigma-Aldrich) and fixed for 10 h at 4 °C, before cells pellets were embedded in paraffin. The sections were deparaffinized, rehydrated and washed with EnVision FLEX wash buffer (Dako). For PD-L2 staining, antigen retrieval was performed using Tris-EDTA buffer (pH 9) at 97 °C for 20 min. After blocking endogenous peroxidase, slides were blocked with 5% goat serum plus 2.5% BSA for 60 min. Slides were then incubated with anti-PD-L2 (catalog no. 82723, Cell Signaling Technology) diluted 1:25 in EnVision FLEX antibody diluent overnight at 4 °C. Next day, slides were incubated with anti-goat horseradish peroxidase (HRP) for 45 min and developed for 10 min by adding 3,3′-diaminobenzidine (DAB). Slides were then dehydrated and mounted with DPX Mountant. For p21 detection, IHC was performed using a Ventana Discovery XT for the p21 clone HUGO 291H/B5 (Spanish National Cancer Research Center), undiluted, for 60 min. Antigen retrieval was performed with Cell Conditioning 1 (CC1) buffer (catalog no. 950-124, Roche). Secondary antibodies used were the OmniMap anti-rat HRP (catalog no. 760-4457, Roche).

To costain PD-L2 with either p21 or cleaved caspase 3 in pellets from human cell lines, IHC was performed using a Ventana Discovery XT for cleaved caspase 3 (Asp175) at 1:300 dilution (catalog no. 9661, Cell Signaling Technology), human p21 (SX118) at 1:100 dilution (catalog no. M7202, Agilent Technologies) and PD-L2 (366C.9E5) at 1:100 dilution (catalog no. MABC1120, Merck Millipore) for 60 min at room temperature. Antigen retrieval was performed with CC1 buffer for p21 and with ULTRA Cell Conditioning 2 buffer (catalog no. 950-223, Roche) for cleaved caspase 3 and PD-L2. Blocking was done with casein (catalog no. 760-219, Roche); the secondary antibody was the OmniMap anti-Rabbit HRP (760-4311, Roche). Rabbit antibody to IgG1 + IgG2a + IgG3 (clone M204-3) at 1:500 dilution for 32 min at room temperature (catalog no. ab133469, Abcam) was used to enhance p21 labeling. Antigen–antibody complexes were revealed with the ChromoMap DAB Kit (catalog no. 760-159, Roche) or Purple Kit (catalog no. 760-229, Roche). In all cases, the PD-L2 antibody protocol was performed after staining for cleaved caspase 3 (Asp175) and for p21. The specificity of staining was confirmed by staining with rabbit IgG (catalog no. NBP2-24891, Novus Biologicals) and mouse IgG1, kappa monoclonal (clone MOPC-21, catalog no. ab18443, Abcam) isotype controls. Digital scanned brightfield images were acquired with a NanoZoomer 2.0-HT C9600 digital scanner (Hamamatsu) equipped with a 20× objective. All images were visualized with a gamma correction set at 1.8 in the image control panel of the NDP.view 2 U12388-01 software (Hamamatsu Photonics).

### Flow cytometry

Cells were collected in PBS with 10 mM EDTA at 37 °C or 0.25% trypsin-EDTA (Invitrogen). Cell viability was assessed using yellow LIVE/DEAD dye solution (Invitrogen) for 15 min at 4 °C and washed before antibody staining or propidium iodide (1:1,000 dilution) was added. Cells were stained with anti-PD-L2-biotin (clone MIH18, Miltenyi Biotec) diluted 1:11 in FACS buffer (0.5% BSA, 2 mM EDTA in PBS) for 15–30 min; after two washes, samples were incubated with anti-biotin-APCVio770 (clone REA746, Miltenyi Biotec) diluted 1:50 in FACS buffer for 15–30 min. After repeated washes, cells were filtered using a 70-μm cell strainer and analyzed on a Gallios flow cytometer (Beckman Coulter), BD LSR Fortessa or BD Celesta and with FlowJo v.10.0.7.

### CRISPR

For the PD-L2 KOs, mouse and human single-guide RNAs (sgRNAs) were designed using the CHOPCHOP web tool (http://chopchop.cbu.uib.no) and cloned into pSpCas9(BB)-2A- Puro (PX459) (catalog no. 48139, Addgene) and lentiCRISPR v2 (catalog no. 52961, Addgene). sgRNA sequences can be found in Supplementary Table 3.

### PCR of genomic DNA

Genomic DNA (gDNA) was isolated using the DNeasy Blood and Tissue Kit (QIAGEN) according to the manufacturer’s instructions. A total of 500 ng of gDNA were added to a mix with 0.2 mM deoxynucleotide triphosphate, 1 μM of primers hybridizing to intronic regions spanning exon 3 of PD-L2 and 1 U of BIOTAQ DNA polymerase (Ecogen). After 35 cycles of amplification, the PCR products were separated using electrophoresis on a 1% agarose gel. PCR bands were gel-purified using a QIAquick Gel Extraction Kit (QIAGEN). Isolated PCR fragments were then sent for sequencing to Eurofins Genomics and analyzed using Serial Cloner v.2.6.1. Primer sequences can be found in Supplementary Table 3.

### Transfection

Cells were transfected with pSpCas9(BB)-2A-Puro (PX459) using FuGENE 6 (Promega) according to the manufacturer’s recommendations. A total of 3 μg of sgRNA containing the PX459 plasmid were transfected. After 3 days, successfully transfected cells were selected using puromycin (Merck Millipore). Cells were then either used in bulk or single clones were isolated by plating 0.5 cells per well in a 96-well plate until colony formation. Successfully generated KOs from single-cell colonies were then assessed by sequencing the sgRNA targeted exons, IHC and flow cytometry for genome editing and PD- L2 protein expression, respectively.

### Generation of lentiviruses and infections

Lentiviruses were produced by transfecting HEK 293T cells with p8.91 (Gag-Pol expressor), pMDG.2 (VSV-G expressor) and lentiCRISPR v2 to generate bulk PD-L2 KO cells or luciferase (catalog no. 105621, Addgene) to monitor in vivo bioluminescence according to standard procedures. Virus batches were collected 48, 72 and 96 h after transfection. Cellular debris was removed by centrifugation and filtering. The supernatant containing the virus was then used fresh or stored using snap-freezing aliquots supplemented with 8 μg ml^−1^ polybrene (Thermo Fisher Scientific) in ethanol or dry ice, which were stored at −80 °C. Recipient cells were incubated for 8 h with lentivirus and selected 3 days later with puromycin (Merck Millipore) or G418 (Thermo Fisher Scientific) for the lentiCRISPR v2 or luciferase experiments, respectively.

### Plasma membrane proteomic screening

Plasma membrane proteomic screening was assayed as described previously^36^. Briefly, SK-MEL-103 cells were induced to senesce by adding 200 nM doxorubicin for 48 h, or 1 μM palbociclib for the duration of the experiment (7 days). Plasma membrane proteins were extracted using a plasma membrane protein extraction kit (catalog no. ab65400, Abcam). Proteins were dissolved in UT buffer (8 M urea, 2 M thiourea, 100 mM Tris-HCl, pH 8.0) and digested using a standard Filter Aided Sample Preparation protocol. The proteins were reduced, alkylated, sequentially digested and desalted. Liquid chromatography–tandem mass spectrometry (MS/MS) was performed by coupling an UltiMate 3000 RSLCnano LC System to either a Q Exactive HF or Q Exactive HF-X mass spectrometer (Thermo Fisher Scientific). MS/MS spectral resolution was set to 15,000 (200 *m/z*). The ion target values were 3 × 10^6^ for MS (maximum injection time of 25 ms) and 1 × 10^5^ for MS/MS (maximum injection time of 22 ms). Raw files were processed with MaxQuant using the standard settings against either a human protein database (UniProtKB/Swiss-Prot, 20,373 sequences). Cysteine carbamidomethylation was set as a fixed modification whereas methionine oxidation and protein N-terminal acetylation were set as variable modifications. Minimal peptide length was set to seven amino acids; a maximum of two tryptic missed cleavages were allowed. Results were filtered at an FDR of 0.01 (peptide and protein level). Afterwards, the proteinGroups.txt file was loaded in ProStaR^49^ using the LFQ intensity values for further statistical analysis. Differential analysis was performed using the empirical Bayesian statistics limma. Proteins with a *P* < 0.05 and a log_2_ ratio greater than 0.58 (1.5 in non-log scale) were defined as upregulated. The FDR was estimated to be below 5%.

### Transcriptomic analysis and gene set enrichment analysis

RNA was extracted from PD-L2 WT and PD-L2 KO Panc02 cells, treated with 200 nM doxorubicin for 48 h or untreated, using the RNeasy Mini Kit (QIAGEN). Libraries for RNA-seq were prepared at the Institute for Research in Biomedicine (IRB) Barcelona Functional Genomics Core Facility using standard operating procedures. Briefly, mRNA was isolated from 1.5 μg of total RNA using the NEBNext Poly(A) mRNA Magnetic Isolation Module (New England Biolabs). Next-generation sequencing libraries were prepared from the purified mRNA using the NEBNext Ultra II RNA Library Prep Kit for Illumina (New England Biolabs). An equimolar pool was prepared and sequenced on a NextSeq 550 (Illumina) at the IRB. A minimum of 32.2 million reads were obtained for all samples. Single-end reads were aligned to the mouse genome version mm10 using STAR (v.2.5.2b). SAM files were converted to BAM files and sorted using sambamba. The count matrix was generated with Rsubread^50^ with the built-in annotation for mm10. DESeq2 (ref. ^51^) was used for differential expression analysis with fold change shrinkage as implemented in the lfcShrink function. Functional enrichment analysis was performed over gene sets defined in the Molecular Signatures Database hallmark gene set collection. The rotation-based approach for enrichment^52^, implemented in the R package limma, was used to represent the null distribution. The minimum–maximum mean enrichment statistic proposed elsewhere, under restandardization, was considered for competitive testing.

### Drug class enrichment analysis

Datasets from the LINCS L1000 project (http://www.lincsproject.org/) were analyzed and differential expression of *PDCD1LG2* was detected by comparing control and treated samples introducing a correction by cell line and batch effect^53^. A total of 4,690 drugs with common names were then further processed and categorized into drugs sets reflecting their mode of action. The resulting drug set enrichment analysis was then compared to a drug set consisting of random drugs.

### Syngeneic Panc02 pancreatic tumors

Syngeneic pancreatic adenocarcinoma Panc02 cells expressing firefly luciferase were orthotopically injected in the pancreas of male, 8–16-week-old immunocompetent C57BL/6 or athymic nude mice (5 × 10^5^ cells in 50 μl PBS). Tumor growth was assessed once a week using an IVIS Spectrum Imaging System (PerkinElmer). Ten minutes after intraperitoneal injection of 75 mg kg^−1^ luciferin, bioluminescence was recorded. Quantification of tumor burden was performed using the Living Image 3.2 software (PerkinElmer). Then, 4 mg kg^−1^ doxorubicin (Sigma-Aldrich), or PBS as vehicle, was applied intraperitoneally in the indicated experimental groups at days 7, 10 and, unless otherwise noted, 24 after surgery. Survival was monitored until the animals reached the humane endpoints related to tumor size, subcutaneous edema or ascites. For the short-term determinations, additional cohorts of mice were euthanized at day 8 or day 12, that is, 24 h or 5 days after the first doxorubicin dose; tumors were formalin-fixed for IHC or processed for mass cytometry. For the coinjection experiments, we injected orthotopically in the pancreas of immunocompetent mice 5 × 10^5^ PD-L2 KO cells in 50 μl PBS, alone or mixed with 5 × 10^5^ senescent cells, either WT or PD-L2 KO, induced to senesce with doxorubicin 200 nM for 48 h and injected on day 7 after the start of treatment.

### Treatments with blocking and depleting antibodies

For selective elimination of immune cell populations in male 8–16-week-old C57BL/6 mice with Panc02 orthotopic tumors, mice were treated twice a week, starting 3 days after surgery, with 100 μg each of, alternatively, anti-CD4 (clone GK1.5, catalog no. BP0003-1, Bio X Cell), anti-CD8α (clone 2.43, catalog no. BE0061, Bio X Cell) or IgG2b (clone LTF-2, catalog no. BP0090, Bio X Cell) as an isotype control. Doxorubicin was administered on days 7 and 10 as described. Tumor growth was monitored by IVIS and mice were euthanized on day 28. Longitudinal depletion of Gr1^+^ cells in mice with Panc02 orthotopic tumors was performed by treatment with 200 μg anti-Gr1 (clone RB6-8C5, catalog no. BE0075, Bio X Cell) or isotype control (clone LTF-2, catalog no. BP0090, Bio X Cell) twice a week, starting 3 days after surgery. Longitudinal blocking of PD-1 was performed by treatment with 200 μg anti-PD-1 (clone RPM1-14, catalog no. BE0146, Bio X Cell) or isotype control (IgG2a isotype, catalog no. BE0089, Bio X Cell), twice a week, starting 3 days after surgery. Doxorubicin was administered as described on days 7, 10 and 24; mice were euthanized on day 35.

### Determination of circulating immune populations using flow cytometry

Approximately 200 μl of total blood of mice treated with doxorubicin and anti-CD4 or anti-CD8 antibodies were extracted using terminal cardiac puncture and kept briefly on ice in a tube containing EDTA. Then, 10 ml of red blood cell lysis buffer (BioLegend) were added and samples were incubated for 5 min at 37 °C. Samples were then centrifuged at 350*g* and washed with PBS twice and in 100 μl FACS buffer once, before additional centrifugation and resuspension in 1:400 CD16/CD32 (BD Fc Block, catalog no. 553142, BD Biosciences) for 15 min at 4 °C. Then, 50 μl of a primary antibody mix were added, containing 1:500 anti-CD45-ApCCy7 (catalog no. 103116, BioLegend), 1:300 anti-CD3-APC (catalog no. 17-0032-80, Thermo Fisher Scientific), 1:400 anti-CD4-PerCP Cy5.5 (catalog no. 550954, BD Biosciences) and 1:200 anti-CD8-FITC (catalog no. 11-0081-82, Thermo Fisher Scientific) for an incubation of 25 min at 4 °C. After the incubations, samples were washed three times with FACS buffer, resuspended in 250 μl FACS buffer with 1 μl 4,6-diamidino-2-phenylindole and analyzed with a Gallios flow cytometer.

### Other syngeneic tumor mouse models

A total of 0.4 × 10^6^ HCmel3 cells were subcutaneously injected into 8–16-week-old male C57BL/6 mice. Four weeks later mice were treated twice weekly with 5 mg kg^−1^ intravenous doxorubicin for a total of three times. A total of 0.2 × 10^6^ B16-OVA WT or B16-OVA PD-L2 KO cells were subcutaneously injected into male C57BL/6 mice. Mice were then treated on days 7 and 10 with intravenous 5 mg kg^−1^ doxorubicin and on day 17 with 5 mg kg^−1^ intraperitoneal doxorubicin. Tumor growth was monitored by caliper measurement and tumor volume was calculated using the formula volume = (length × width^2^)/2. Mice were euthanized 2 days after the last treatment.

### SK-MEL-103 xenograft tumors

A total of 10^6^ SK-MEL-103 cells, either PD-L2 WT or PD-L2 KO, were collected, resuspended in a volume of 100 μl PBS/Matrigel and injected in both flanks of 8–16-week-old female athymic nude mice. Once their tumors reached 100–150 mm^3^ (approximately at days 7–10), mice were distributed evenly between the palbociclib-treated group, which received 100 mg kg^−1^ palbociclib by oral gavage in 50 mM sodium lactate every day, or the control group, which received vehicle. Tumor size was monitored for 10 days using a caliper as described above. Tumors were extracted and flash-frozen for further analysis of gene expression or whole-mount SA-β-gal staining, and formalin-fixed for PD-L2 staining as described above.

### IHC in Panc02 pancreatic tumors

Samples were fixed overnight at 4 °C with neutral-buffered formalin (Sigma-Aldrich). Paraffin-embedded tissue sections (2–3-μm-thick) were air-dried and further dried at 60 °C overnight. For p21 detection, IHC was performed using a Ventana Discovery XT for the p21 clone HUGO 291H/B5, undiluted, for 60 min. Antigen retrieval was performed with CC1 buffer. The secondary antibody used was the OmniMap anti-rat HRP. For Ly6G/C (Gr1) staining, IHC was performed using a Ventana Discovery XT for Ly6G/C (catalog no. NB600-1387, Novus Biologicals) primary antibody at 1:100 dilution for 60 min. Antigen retrieval was performed with CC1 buffer. After incubation with the primary antibody, the rabbit anti-rat IgG (catalog no. AI-4001, Vector Laboratories) at 1:500 dilution for 32 min was used followed by the secondary antibody OmniMap anti-rabbit HRP. Antigen–antibody complexes were revealed with the ChromoMap DAB Kit. The specificity of staining was confirmed with the rat IgG isotype control (catalog no. 6-001-F, R&D Systems). For CD3 detection, IHC was performed using an Autostainer Plus (Agilent Technologies). Before IHC, sections were dewaxed as part of the antigen retrieval process using the High pH EnVision FLEX Target Retrieval Solutions (Dako) for 20 min at 97 °C using a PT Link (Agilent Technologies). Quenching of endogenous peroxidase was performed by 10-min incubation with Peroxidase-Blocking Solution (code no. REAL S2023, Dako). Unspecific unions were blocked using 3% goat normal serum (catalog no. 16210064, Thermo Fisher Scientific) for 60 min. Primary antibody FLEX Polyclonal Rabbit Anti-Human CD3 (catalog no. IR503, Agilent Technologies) was diluted 1:10 with Dako antibody diluent (code no. S0809, Agilent Technologies) for 120 min at room temperature. The secondary antibody used was a BrightVision Poly-HRP-Anti Rabbit IgG Biotin-free, ready to use (catalog no. DPVR-110HRP, Immunologic) incubated for 45 min. Antigen–antibody complexes were revealed with 3,3′-diaminobenzidine (code no. K3468, Dako), with the same time exposure per antibody (5 min). Sections were counterstained with hematoxylin (catalog no. S202084, Dako) and mounted with Mounting Medium, Toluene-Free (code no. CS705, Dako) using a Dako CoverStainer. Brightfield images were acquired with a NanoZoomer 2.0-HT C9600 digital scanner equipped with a 20× objective. All images were visualized with a gamma correction set at 1.8 in the image control panel of the NDP.view 2 U12388-01 software. Automated quantification was performed using QuPath^54^.

### SA-β-gal staining

For the SA-β-gal staining of adherent cells, optimal cutting temperature (OCT) compound-embedded tumors or whole-mount tumors were used with homemade solutions adapted from the original protocol^55^. Samples were fixed using a solution containing 5 mM EGTA, 2 mM MgCl_2_ and 0.2% glutaraldehyde in 0.1 M phosphate buffer (pH 7) for 15 min at room temperature (whole-mount tumors, 45 min). After washing with PBS, samples were stained with a solution containing 40 mM citric acid, 5 mM potassium cyanoferrate (II), 5 mM potassium cyanoferrate (III), 150 mM sodium chloride and 2 mM magnesium chloride in 0.1 M phosphate buffer (pH 6), with 1 mg ml^−1^ 5-bromo-4-chloro-3-indolyl ß-D-galactoside (X-Gal) at 37 °C overnight or for 16 h. For OCT compound-embedded, frozen tumors, 8–10-μm-thick sections were obtained, followed by fixing and staining as described above. Nuclear Fast Red was used as a counterstain.

### Identification of immune cell populations using mass cytometry

Single-cell suspensions were prepared from Panc02 pancreatic tumors. Briefly, tumors were manually minced and tumor fragments were incubated in 10 mg ml^−1^ collagenase I and DNase in DMEM for 30 min at 37 °C in gentleMACS C tubes, with several processing steps in a gentleMACS dissociator according to the manufacturer’s instructions. The cell suspension was passed through a 70-μm strainer, washed in PBS and incubated in red blood cell lysis buffer (BioLegend). Cells were then incubated with anti-mouse CD16/CD32 at 1:400 dilution (BD Fc Block) for 10 min at 4 °C in Maxpar Cell Staining Buffer. Then, an equal volume of the 16-antibody cocktail from the Maxpar Mouse Sp/LN Phenotyping Panel Kit (catalog no. 201306, Fluidigm) was added and incubated for 30 min. Anti-mouse PD-1 (J43) ^159^Tb (catalog no. FLU3159023) was used to detect PD-1^+^ cells. After two washes with 2 ml Maxpar Cell Staining Buffer, cells were fixed in 1.6% formaldehyde in PBS for 10 min at room temperature. Samples were then centrifuged at 800*g* for 5 min and resuspended in 125 nM Cell-ID Intercalator-Ir (Fluidigm) in Maxpar FIX and PERM Buffer (Fluidigm) and incubated at 4 °C overnight. Acquisition was performed the following day after washing with Cell Acquisition Solution (Fluidigm) and mixing with diluted EQ bead solution (Fluidigm), according to the manufacturer’s instructions, in a Helios detector (Fluidigm). The gating strategy was fully standardized and is described in detail by the manufacturer. Briefly, Intercalator-Ir^+^ cells were selected, from which CD19^+^B220^+^ B lymphocytes were defined. Next, T cell receptor-β (TCR-β)^+^CD3^+^ T cells were selected and, within this population, CD4^+^ and CD8^+^ T cells were detected. From the TCR-β^−^CD3^−^ population, sequentially, we identified by sequential exclusion NK1.1^+^ NK cells and CD11c^+^ dendritic cells. Finally, among the CD11b^+^ cells, macrophages and MDSCs were identified depending on Gr1 positivity.

### Measurement of intratumoral cytokine and chemokine levels

Panc02 pancreatic tumors were flash-frozen after excision; approximately 10 mg of tumor mass was homogenized in radioimmunoprecipitation assay (RIPA) lysis buffer (Merck Millipore) using a FastPrep-24 5G homogenizer (MP Biomedicals) in the presence of the Halt protease inhibitor cocktail (Thermo Fisher Scientific). Protein concentration was determined using a colorimetric assay (DC protein assay kit, Bio-Rad Laboratories). All samples were diluted to a protein concentration of 1.2 mg ml^−1^ in a total volume of 75 μl RIPA and shipped to Eve Technologies (Mouse Cytokine Array/Chemokine Array 31-Plex, Eve Technologies). For the cytokines and chemokines included in the assay but not shown in the manuscript (IL-13, IL-12p40, CCL4), no changes were observed.

### MMTV-PyMT model

Male PyMT mice were mated with FVB WT female mice to generate female littermates that were PyMT WT. Mice were genotyped for the PyMT allele using PCR with the following primers: forward 5′-CGCACATACTGCTGGAAGAA and reverse 5′-TGCCGGGAACGTTTTATTAG. Weights were recorded for every experiment. Mice were housed in a specific pathogen-free environment. At 9 weeks of age, PyMT mice were palpated to detect the onset of mammary tumor development. Mice were then injected intraperitoneally with 4 mg kg^−1^ doxorubicin or an equivalent volume of PBS, once a week for 4 weeks. Additional groups of mice were treated with anti-mouse PD-L2 (clone TY25, catalog no. BE0112, Bio X Cell), alone or in combination with doxorubicin. The rest of doxorubicin-treated mice received rat IgG (IgG2a isotype). From the detection of palpable tumors, mice were monitored for tumor growth with a caliper twice a week. Total tumor volume was determined using the formula volume = (length × width^2^)/2. Mice were euthanized 4 weeks after the onset of tumors and the start of the treatments. To deplete specific immune populations, mice were injected intraperitoneally with anti-CD4 (clone YTS 191, catalog no. BE0119, Bio X Cell), anti-CD8α (clone 53-6.7, catalog no. BE0004-1, Bio X Cell) or rat IgG (IgG2a isotype) and euthanized at week 13. All antibody treatments were administered intraperitoneally, every 3 days for 4 weeks, at a 10 mg kg^−1^ dose.

### IHC and SA-β-gal in mammary tumors

For PyMT mice, mammary glands were either formalin-fixed or whole-mounted, as described previously, and paraffin-embedded. The IHC experiments were performed using the Dako EnVision+ System-HRP Kit. Using the Leica microtome system, 3-μm-thick sections were obtained; antigen retrieval was performed using the Tris-EDTA buffer (pH 9) by boiling followed by washing in running water and blocking for endogenous peroxidase for 15 min at room temperature. Slides were washed with Tris-buffered saline; after a 1-h incubation with 3% BSA blocking solution, each slide was incubated with primary antibody against PD-L2 (1:100 dilution, catalog no. 49189, Cell Signaling Technology), CD3 (1:100 dilutions, catalog no. ab16669, Abcam), CD4 (1:1,000 dilution, catalog no. ab183685, Abcam) or CD8 (1:2,000 dilution, catalog no. ab217344, Abcam) at 4 °C overnight. After washing, slides were incubated with System-HRP Labelled Polymer Anti-Rabbit (catalog no. K400311-2, Dako) secondary antibody for 1 h at room temperature. Tissues were counterstained with hematoxylin after the substrate (DAB) reaction step. The liver and myocardium of control and anti-PD-L2-treated mice were fixed and paraffin-embedded as described, then stained with hematoxylin and eosin. For SA-β-gal staining, mammary gland samples were whole-mounted using a fixative solution of 0.2% glutaraldehyde and 2% paraformaldehyde in PBS. Overnight staining with senescence β-galactosidase Staining Kit (Cell Signaling Technology) was performed directly in the fixed samples, before being dipped in paraffin. Using the Leica microtome system, 3-μm-thick sections were obtained and counterstained with Liquid-Stable Nuclear Fast Red (AMRESCO).

### Ethical oversight of tumor size in experimental mice

Maximum tumor sizes according to the ethics committees were: (1) 1,500 mm^3^ for SK-MEL-103 xenograft tumors. This limit was not surpassed by any animal; (2) 1,000 mm^3^ for other subcutaneous tumor models. This limit was not surpassed by any animal; (3) 1,200 mm^3^ for mammary tumors. Animals were euthanized immediately before reaching this limit or, exceptionally, as soon as a tumor surpassing the limit was detected. Maximum tumor size in practice was, in any case, 1,300 mm^3^; (4) for orthotopic pancreatic tumors, the humane endpoint was determined by veterinary decision after evaluation of the external aspect of the animal. Veterinary oversight was increased to a daily frequency as soon as luminescence reached 10^9^ p s^−1^. Maximum tumor luminescence was, in any case, 5 × 10^9^ p s^−1^.

### Human samples

Tumor specimens were obtained from one patient with metastatic head and neck cancer (VHIO-008), one patient with melanoma (VHIO-088) and one patient with endometrial cancer (VHIO-35035). Patients VHIO-008 and VHIO-088 were refractory to standard lines of therapy before sample procurement. Patient VHIO-35035 did not receive any treatment before sample procurement. All samples were obtained from patients enrolled in two studies approved by the Vall d’Hebron Hospital ethics committee (PR(AG)252/2016, PR(AG)537/2019). Written informed consent was obtained from all individuals. Tumor cell lines were established by culturing one tumor fragment in RPMI 1640 supplemented with 20% human AB serum (Biowest), 100 U ml^−1^ penicillin, 100 μg ml^−1^ streptomycin, 25 mM HEPES (Thermo Fisher Scientific), 10 μg ml^−1^ gentamicin and 1.25 μg ml^−1^ amphotericin B at 37 °C and 5% CO_2_. After 1 month, adherent and non-adherent cells were cultured in RPMI 1640 supplemented with 20% FCS (HyClone), 100 U ml^−1^ penicillin, 100 μg ml^−1^ streptomycin, 25 mM HEPES (Thermo Fisher Scientific), 10 μg ml^−1^ gentamicin (Lonza) and 1.25 μg ml^−1^ amphotericin B (Gibco). Occasionally, we used differential trypsinization to enrich for epithelial cells. The medium was changed once a month until the tumor cell line was established. Tumor cell lines were authenticated using whole-exome sequencing.

### Cell treatments, SA-β-gal assay and analysis of mRNA levels in human primary samples

To induce senescence, cells were treated with the DNA-damaging agent bleomycin (12 mU l^−1^, catalog no. B8416, Sigma-Aldrich) for 48 h and assayed 7 days later. For SA-β-gal, cells were washed with PBS, fixed with 0.2% glutaraldehyde for 10 min, washed with PBS and incubated overnight at 37 °C with a staining solution containing 1 mg ml^−1^ X-Gal (catalog no. MB1001, Melford BioLaboratories) prepared in dimethylformamide (catalog no. D4551, Sigma-Aldrich) at pH 6. Cells were then washed in PBS and visualized using a Nikon Eclipse TS2 brightfield microscope. To analyze gene expression, total RNA was extracted with TRIzol (Invitrogen) according to the manufacturer’s protocol. Gene expression was analyzed by qPCR with reverse transcription using the PowerUp SYBR Green Master Mix (Thermo Fisher Scientific) in the 7900HT Fast Real-Time PCR System (Applied Biosystems). Cycle threshold values were normalized to GAPDH. Primer sequences can be found in Supplementary Table 3.

### Statistics and reproducibility

Results are presented as the mean ± s.e.m. for all data. All statistical analyses were performed using Prism 8 (GraphPad Software); *P* < 0.05 was considered significant. Tests were applied as described in the figures; two-tailed Student’s *t*-tests were used for pairwise comparisons between two isolated conditions, while a one-way ANOVA with Tukey’s post-hoc test for multiple comparisons were used for experiments with three or more experimental conditions. For datasets including two independent factors, where one of them was usually time, a two-way ANOVA was used. Specific tests for select experiments are detailed in the figure legends or in the methodological description. Data distribution was assumed to be normal and was formally tested for the in vivo experiments.

No statistical method was used to predetermine sample size. No data were excluded from the analyses. The experiments were not randomized. Mice were age-matched and tumor size was distributed evenly whenever possible between groups. For surgeries in mice, the operator was blinded to the cell type being injected. Quantification of IHC images was performed in a blinded fashion. The in vitro experiments were not performed in a blinded fashion.

### Reporting summary

Further information on research design is available in the Nature Portfolio Reporting Summary linked to this article.

## Supplementary information

Reporting Summary

Supplementary Tables Supplementary Tables 1–3.

## Source data

Source Data Fig. 1 Primary data for statistics.

Source Data Fig. 1 Representative Images.

Source Data Fig. 2 Primary data for statistics.

Source Data Fig. 3 Primary data for statistics.

Source Data Fig. 4 Primary data for statistics.

Source Data Fig. 5 Primary data for statistics.

Source Data Extended Data Fig. 1 Primary data for statistics.

Source Data Extended Data Fig. 2 Primary data for statistics.

Source Data Extended Data Fig. 3 Primary data for statistics.

Source Data Extended Data Fig. 4 Primary data for statistics.

Source Data Extended Data Fig. 6 Primary data for statistics.

Source Data Extended Data Fig. 7 Primary data for statistics.

Source Data Extended Data Fig. 8 Primary data for statistics.

Source Data Extended Data Fig. 9 Primary data for statistics.

## Data Availability

The proteomic screening data are available at ProteomeXchange (http://proteomecentral.proteomexchange.org/cgi/GetDataset) under accession no. PXD033714. The RNA-seq data are available at the Gene Expression Omnibus (accession no. GSE210334). Source data files have been provided for all applicable figures and extended data figures. All other data supporting the findings of this study are available from the corresponding authors upon reasonable request. Source data are provided with this paper.
