# Author Correction: The efficacy of chemotherapy is limited by intratumoral senescent cells expressing PD-L2

**DOI:** 10.1038/s43018-026-01191-6

**Published:** 2026-05-29

**Authors:** Selim Chaib, José Alberto López-Domínguez, Marta Lalinde-Gutiérrez, Neus Prats, Ines Marin, Olga Boix, Andrea García-Garijo, Kathleen Meyer, María Isabel Muñoz, Mònica Aguilera, Lidia Mateo, Camille Stephan-Otto Attolini, Susana Llanos, Sandra Pérez-Ramos, Marta Escorihuela, Fatima Al-Shahrour, Timothy P. Cash, Tamara Tchkonia, James L. Kirkland, María Abad, Alena Gros, Joaquín Arribas, Manuel Serrano

**Affiliations:** 1 Institute for Research in Biomedicine, Barcelona Institute of Science and Technology, Barcelona, Spain; 2 Division of General Internal Medicine, Mayo Clinic, Rochester, MN USA; 3 Vall d’Hebron Institute of Oncology, Vall d’Hebron Barcelona Hospital Campus, Barcelona, Spain; 4 Genentech, South San Francisco, CA USA; 5 Cambridge Institute of Science, Altos Labs, Cambridge, UK; 6 DNA Replication Group, Spanish National Cancer Research Center, Madrid, Spain; 7 Bioinformatics Unit, Spanish National Cancer Research Center, Madrid, Spain; 8 Rejuveron Senescence Therapeutics, Zürich, Switzerland; 9 Department of Physiology and Biomedical Engineering, Mayo Clinic, Rochester, MN USA; 10 Cancer Research Program, Hospital del Mar Medical Research Institute, Centro de Investigación Biomédica en Red Cáncer, Barcelona, Spain; 11 Department of Medicine and Life Sciences, Universitat Pompeu Fabra, Barcelona, Spain; 12 Institució Catalana de Recerca i Estudis Avançats, Barcelona, Spain

**Keywords:** Cancer, Senescence

Correction to: *Nature Cancer* 10.1038/s43018-023-00712-x, published online 24 January 2024.

In the version of this article initially published, the representative images for the control and 5 μM palbociblib treatments of SK-MEL-103 cells in Fig. 1a corresponded to control and 1 μM palbociclib-treated cells that we had previously discussed in another article (Llanos, S. et al. *Oncogene* **38**, 3886–3902 (2019); 10.1038/s41388-019-0695-8). This error occurred during figure preparation because the images were selected from a folder containing several independent experiments of control and 1 μM and 5 μM palbociclib treatment of SK-MEL-103 cells. The correct control and 5 μM images have now been included in Fig. 1a and an additional Source Data file was prepared with representative images for each experimental condition from independent experiments. The amended figure and new Source Data are now available in the HTML and PDF versions of the article.

