# Supplementary material for: The efficacy of chemotherapy is limited by intratumoral senescent cells expressing PD-L2
Source: Nat Cancer. 2024 Jan 24;5(3):448–62. doi: 10.1038/s43018-023-00712-x (PMC10965441; doi:10.1038/s43018-023-00712-x)
Supplement: Supplementary file 1 [file 43018_2023_712_MOESM1_ESM.pdf]

nature portfolio

Corresponding author(s): Manuel Serrano

Last updated by author(s): Dec 13, 2023

Reporting Summary

Nature Portfolio wishes to improve the reproducibility of the work that we publish. This form provides structure for consistency and transparency in reporting. For further information on Nature Portfolio policies, see our Editorial Policies and the Editorial Policy Checklist.

Statistics

For all statistical analyses, confirm that the following items are present in the figure legend, table legend, main text, or Methods section.

n/a	Confirmed	
☐	☒	The exact sample size (*n*) for each experimental group/condition, given as a discrete number and unit of measurement
☐	☒	A statement on whether measurements were taken from distinct samples or whether the same sample was measured repeatedly
☐	☒	The statistical test(s) used AND whether they are one- or two-sided<br>*Only common tests should be described solely by name; describe more complex techniques in the Methods section.*
☐	☒	A description of all covariates tested
☐	☒	A description of any assumptions or corrections, such as tests of normality and adjustment for multiple comparisons
☐	☒	A full description of the statistical parameters including central tendency (e.g. means) or other basic estimates (e.g. regression coefficient) AND variation (e.g. standard deviation) or associated estimates of uncertainty (e.g. confidence intervals)
☐	☒	For null hypothesis testing, the test statistic (e.g. *F*, *t*, *r*) with confidence intervals, effect sizes, degrees of freedom and *P* value noted<br>*Give P values as exact values whenever suitable.*
☒	☐	For Bayesian analysis, information on the choice of priors and Markov chain Monte Carlo settings
☒	☐	For hierarchical and complex designs, identification of the appropriate level for tests and full reporting of outcomes
☒	☐	Estimates of effect sizes (e.g. Cohen's *d*, Pearson's *r*), indicating how they were calculated

Our web collection on statistics for biologists contains articles on many of the points above.

Software and code

Policy information about availability of computer code

Data collection	Flow cytometry: Gallios flow cytometer (Beckman Coulter), BD LSR Fortessa and BD Celesta.<br>IHC: NanoZoomer-2.0 HT C9600 digital scanner<br>Bioluminescence: IVIS Spectrum Imaging System (Perkin Elmer)<br>Sequencing: NextSeq550 (Illumina)
Data analysis	QuPath 0.4.3, GraphPad Prism 8, FlowJo (v10), Living Image 3.2, LSTAR (v 2.5.2b) MaxQuant (2.2), Serial Cloner (2.6.1.), sambamba-0.8.2, Rsubread_2.4.3, DESeq2_1.30.1, limma_3.46.0. MSigDB genesets (GO BP, CC, MF and KEGG pathways) were retrieved from the Bioconductor org.Hs.eg.db package (v 3.0.0)

For manuscripts utilizing custom algorithms or software that are central to the research but not yet described in published literature, software must be made available to editors and reviewers. We strongly encourage code deposition in a community repository (e.g. GitHub). See the Nature Portfolio guidelines for submitting code & software for further information.

Data

Policy information about availability of data

All manuscripts must include a data availability statement. This statement should provide the following information, where applicable:

- Accession codes, unique identifiers, or web links for publicly available datasets
- A description of any restrictions on data availability
- For clinical datasets or third party data, please ensure that the statement adheres to our policy

The proteomic screen data is available at ProteomeXchange, project accession: PXD033714. The RNA sequencing data has been uploaded to Gene Expression Omnibus (GEO), accession number: GSE210334. Publicly available data was obtained from LINCS L100 project public database and Molecular Signatures Database (MSigDB) hallmark gene set collection.

Human research participants

Policy information about studies involving human research participants and Sex and Gender in Research.

Reporting on sex and gender	We did not seek or obtain access to sex or gender information for the human samples to which we had access.
Population characteristics	Tumour specimens were obtained from one patient with metastatic head and neck cancer, VHIO-008, one melanoma, VHIO-088, and one endometrial cancer, VHIO-35035. Patients VHIO-008 and VHIO-088 were refractory to standard lines of therapy prior to sample procurement. Patient VHIO-35035 did not receive any treatment prior to sample obtention. No covariate-relevant characteristics apply to this samples since no population-wide or statistical analysis has been applied other than specific parameters within each individual human sample.
Recruitment	Informed consent was obtained from all subjects, but no recruitment process was necessary for this work. We obtained access to available samples from the clinical trials PR(AG)252/2016 and PR(AG)537/2019, which were unrelated to this research.
Ethics oversight	All samples were obtained from patients enrolled in two studies approved by the Vall d'Hebron Hospital ethical committee [PR(AG)252/2016, PR(AG)537/2019].

Note that full information on the approval of the study protocol must also be provided in the manuscript.

Field-specific reporting

Please select the one below that is the best fit for your research. If you are not sure, read the appropriate sections before making your selection.

☒ Life sciences ☐ Behavioural & social sciences ☐ Ecological, evolutionary & environmental sciences

For a reference copy of the document with all sections, see nature.com/documents/nr-reporting-summary-flat.pdf

Life sciences study design

All studies must disclose on these points even when the disclosure is negative.

Sample size	No statistical methods were used to predetermine sample size estimates. For in vitro experiments sample size was determined based on preliminary experiments. For in vivo experiments, sample size was determined based on previous experiments and small pilot studies.
Data exclusions	No data were excluded from the analysis.
Replication	All in vitro experiments were successfully replicated at least in three different experiments. In vivo studies have been performed as follows. B16-OVA tumour model has been performed once since it was designed to test whether our findings with the Panc02 model could also apply to other tumour models, and it was performed with high number of animals (n = 11). The experiment in Fig 2a has been performed at least two times in independent experiments.
Randomization	Animals were randomized to ensure similar tumour sizes in treatment groups. For human samples, just individual samples from 4 patients were analyzed, one per tumour type upon recruitment and depending on the viability of the primary tissue, so randomization was not applicable.
Blinding	For surgeries in mice, the operator was blind to the cell type being injected. Quantification of IHC and IF images was performed in a blind fashion. In vitro experiments the authors were not blinded since a single operator was responsible for the treatment and for data analysis, and because senescence-inducing treatments induced evidence morphological changes in the tissue cultures.
