# Supplementary material for: The efficacy of chemotherapy is limited by intratumoral senescent cells expressing PD-L2
Source: Nat Cancer. 2024 Jan 24;5(3):448–62. doi: 10.1038/s43018-023-00712-x (PMC10965441; doi:10.1038/s43018-023-00712-x)
Supplement: Supplementary file 2 [file 43018_2023_712_MOESM4_ESM.pdf]

Source Data figure 1

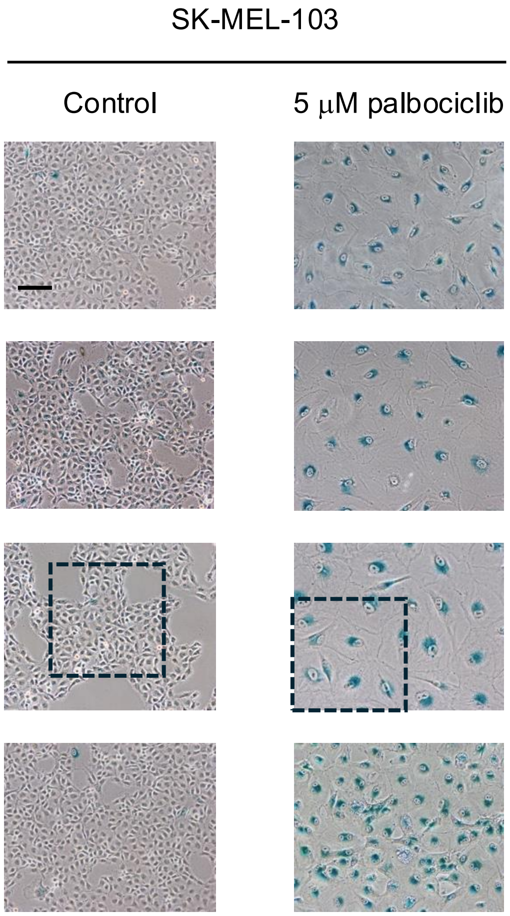

Source Data figure 1. Representative images of several experiments showing untreated SK-MEL-103 cells and cells treated 5 μM palbociclib. The fields depicted in Fig1a are highlighted with a dashed line. Bar = 50 μm.
